# Supplementary material for: Light-mediated communication in responsive materials ranging from individual self-oscillators to feedback-driven network
Source: Nat Commun. 2025 Nov 20;16:11315. doi: 10.1038/s41467-025-66395-3 (PMC12722257; doi:10.1038/s41467-025-66395-3)
Supplement: Supplementary file 1 — Supplementary Information [file 41467_2025_66395_MOESM1_ESM.pdf]

Supplementary Materials for

Light-mediated communication in responsive materials ranging from individual self-oscillators to feedback-driven network

Hongshuang Guo,[1] Kai Li,[2]* Jianfeng Yang[1], Dengfeng Li,[1] Fan Liu,[1] Hao Zeng[1]*

Affiliation:

[1] Faculty of Engineering and Natural Sciences, Tampere University, P.O. Box 541, FI-33101 Tampere, Finland.

[2] Department of Civil Engineering, Anhui Jianzhu University, Hefei 230601, China.

*Correspondence to: kli@ahjzu.edu.cn; hao.zeng@tuni.fi.

This PDF file includes:

1. Supplementary Figures 1-32.

2. Supplementary Notes.

2.1 Single oscillator.

2.2 Coupled oscillators.

2.3 Comparison between single and coupled oscillators

2.4 Sensing and control.

2.5 Scalability – a future plan.

3. References

4. Captions for Supplementary Movies 1-9.

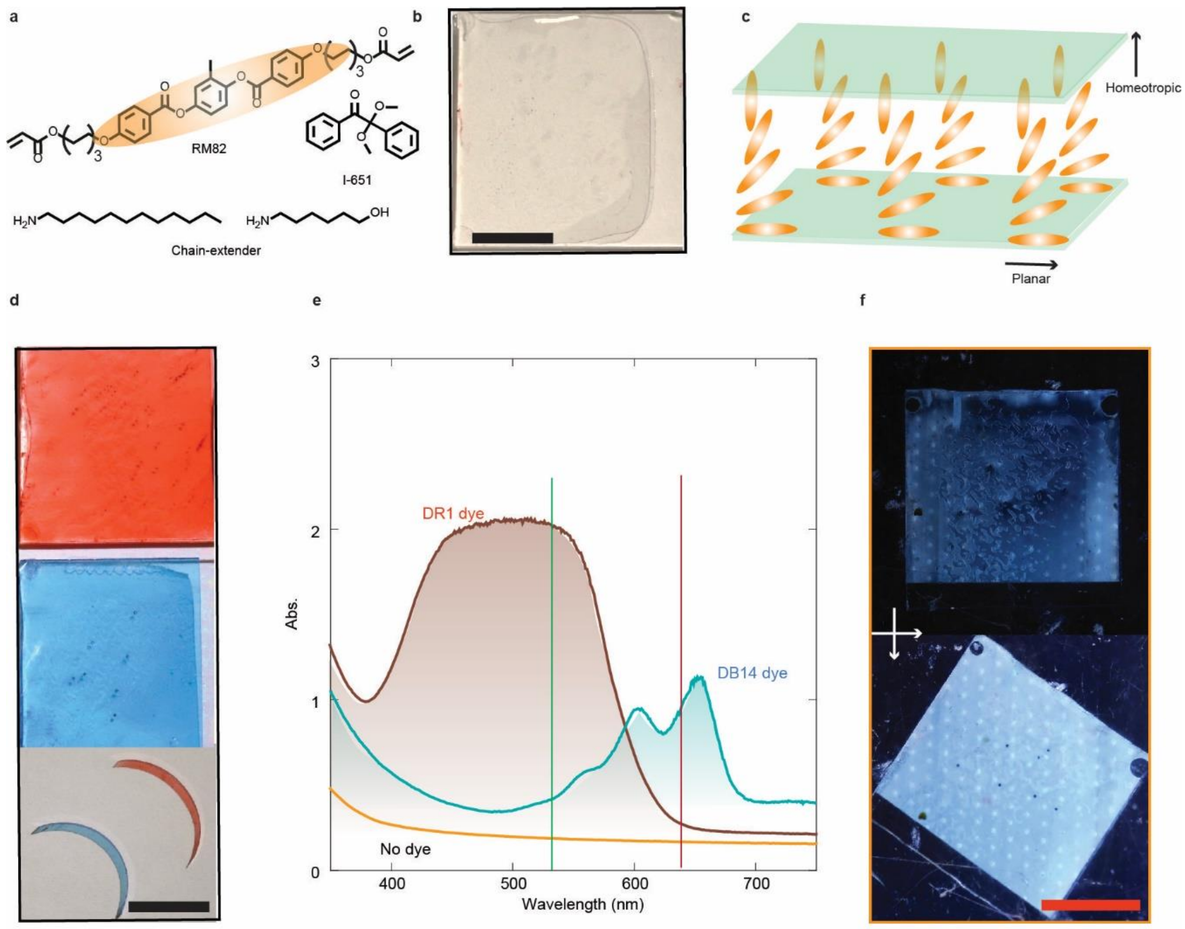

Supplementary Figure 1. Sample preparation. (a) Chemical structures of molecules utilized in LCE synthesis. (b) Photographs displaying a dye-free polymerized LCE film on top of a glass substrate. (c) Schematic drawing shows the splayed orientation of molecules inside the LCE. The arrows denote the types of surface treatment and the corresponding liquid crystal alignment at the surfaces. (d) Photographs displaying an LCE film thermally diffused with Dispersed Red 1 dye (top), Dispersed Blue 14 dye (middle), and strip-like actuators cut from corresponding films (bottom). (e) Absorption spectra of the LCE film before and after dye diffusion. The green line represents the wavelength of the 532 nm laser, red line for the 635 nm laser, utilized for sample excitations. (f) Cross-polarized macroscopic images of a dye-free LCE film. Film thickness: 100 µm. All scale bars are 1 cm.

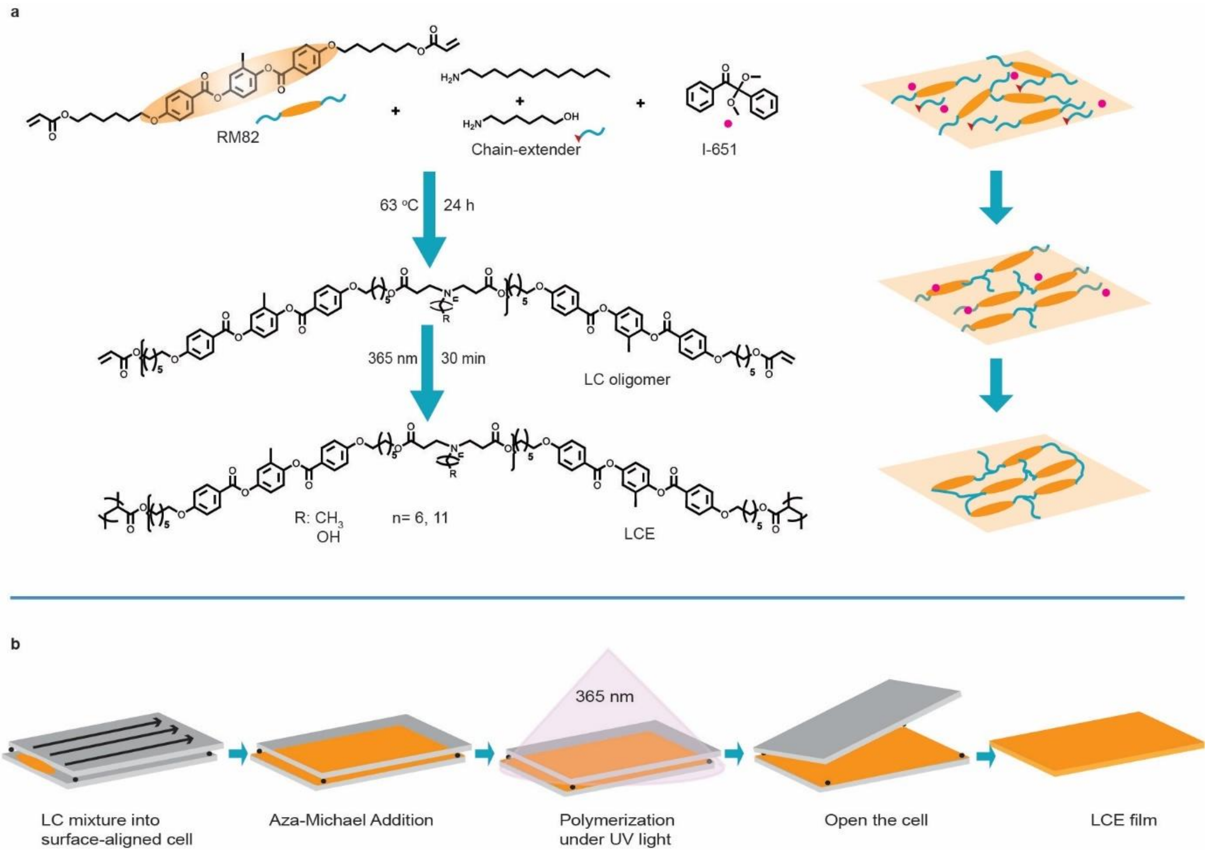

Supplementary Figure 2. LCE synthesis process. (a) The steps of the polymerization reaction (left) and a schematic diagram illustrating the polymerization process (right). (b) Liquid crystalline cell fabrication steps. First, RM82, 6-Amino-1-hexanol, dodecylamine, and 2,2-Dimethoxy-2-phenylacetophenone are homogeneously mixed and filled into a cell at 95°C. Secondly, the cell is placed in an oven at 63°C for 24 hours to conduct Aza-Michael addition-based step-growth polymerization, resulting in the formation of the LC oligomer. Thirdly, the remaining diacrylate end-groups are cross-linked upon UV exposure to form the final LCE network. Finally, the cell is opened by using a blade, and the LCE film actuator is removed from the glass substrate.

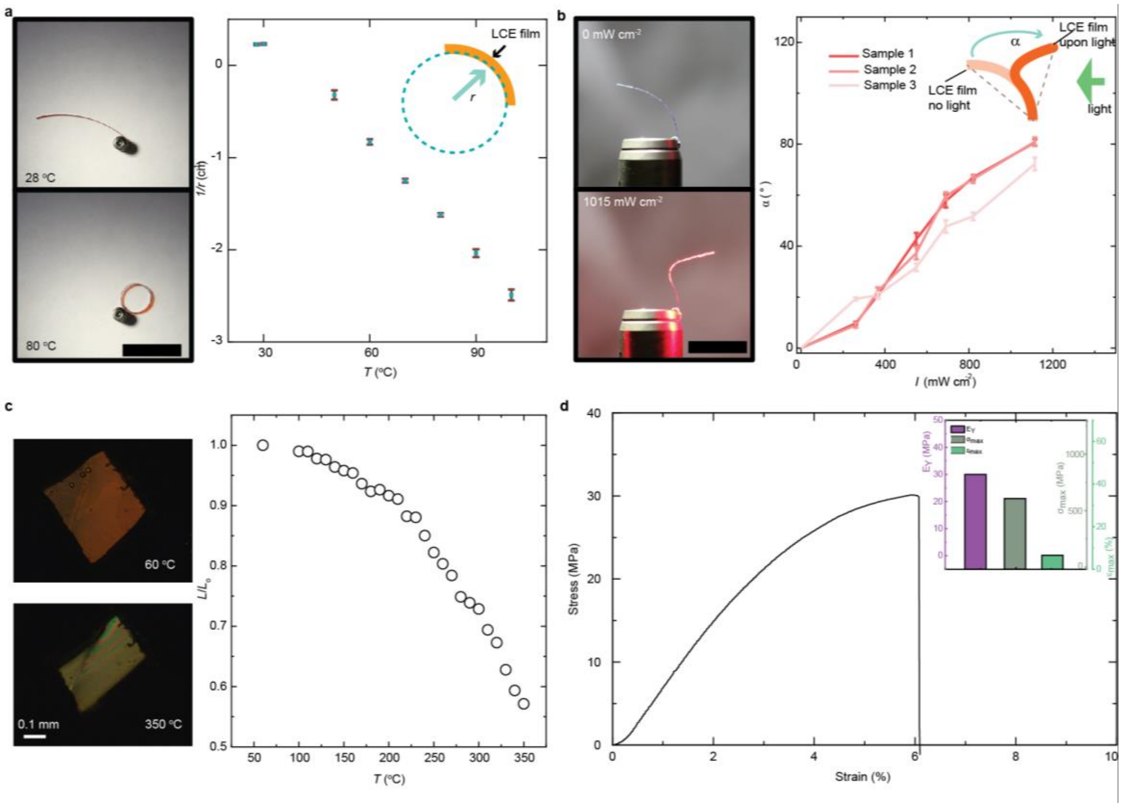

Supplementary Figure 3. Stimuli-responsiveness of LCE actuator. (a) Left: Photographs showing the LCE strip geometries at room condition and elevated temperature. Strip size: 15 × 1 × 0.1 mm^3. The sample is placed on top of a hot plate and covered with a transparent glass window to attain homogeneous temperature distribution. Right: The curvature variation upon increasing the temperature. Curvature is defined as $1/r$, where r is the radius of the strip, as shown in the inset. (b) Left: Photographs displaying the LCE strip geometries upon different illuminating intensities. Strip size: 15 × 1 × 0.1 mm^3. Right: The bending angle of three independent LCE actuators upon change of illuminating light intensity I. The strips are cut from the same LCE film. The bending angle (α) is indicated in the inset. Error bars represent s.d. for n = 3 measurements. The same sample was measured repeatedly. Scale bars in (a, b) are 1 cm. (c) Left: Polarized microscopy images of an LCE film at 60 and 350 °C. Strain curve of LCE upon elevated temperature. L, the length of the film after deformation, L_0, the original length of the LCE. (d) Tensile testing of an LCE strip. Inset shows the measurements of Young's modulus (E_Y), fracture strain (ε_{max}), and tensile strength (σ_{max}).

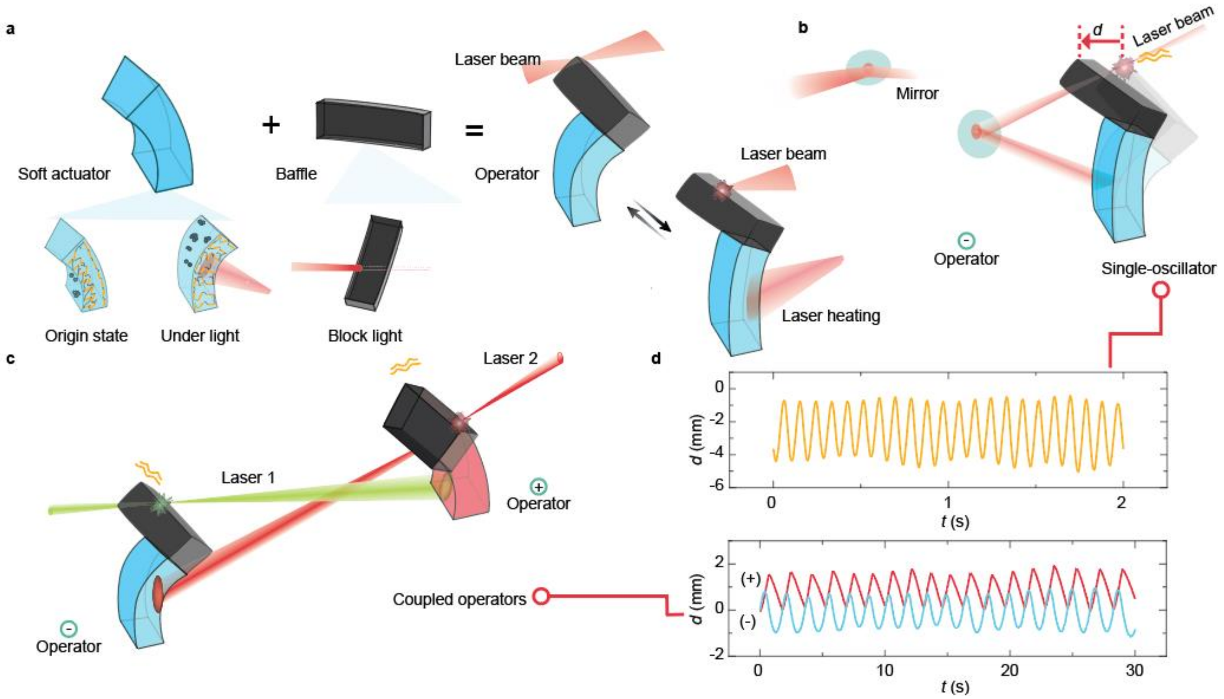

Supplementary Figure 4. Structure assembly for coupled oscillator systems. (a) Schematics showing the assembly of the baffle and LCE actuator to form the (-)operator system. (b) Schematics showing the (-)operator controls the propagation of a light beam that has been reflected by a mirror onto the actuator. This forms a negative feedback self-oscillator. *d*, displacement of the baffle tip position. (c) Schematic diagram illustrating the optical setup to achieve the coupling between a negative and a positive operator. (d) Oscillation data of the single self-oscillator and coupled oscillators under constant light beam excitation. Light for single oscillation: 532 nm, continuous laser, 420 mW, spot size is 2 mm. For coupled oscillators, laser 1: 280 mW, 532 nm, spot, 2 mm, laser 2: 320 mW, 635 nm, spot, 3 mm. LCE sample dimensions are $24 \times 2 \times 0.1$ mm^3, baffle, $5 \times 20 \times 0.01$ mm^3.

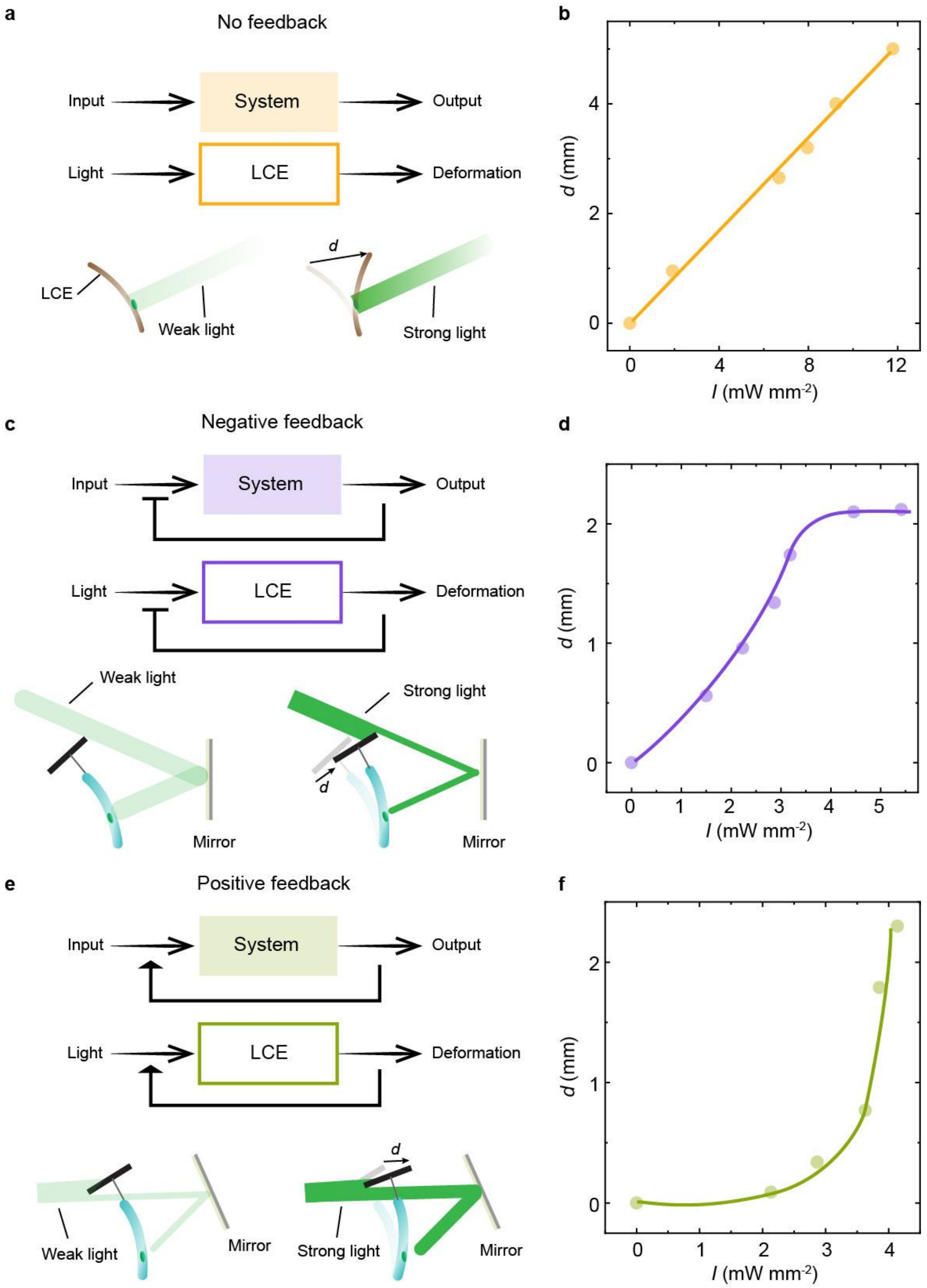

Supplementary Figure 5. Feedback in optomechanical system. (a) Schematics of an LCE actuator without feedback. (b) Characteristic curve of tip displacement upon varying the input light intensity with no feedback. (c) Schematics of a baffle-actuator system with negative feedback in photomechanical response. (d) Characteristic curve of tip displacement regulated by negative feedback in response to changes in light input. (e) Schematics of a baffle-actuator system with positive feedback in photomechanical response. (f) Characteristic curve of tip displacement regulated by positive feedback in response to changes in light input. LCE sample dimensions: $24 \times 2 \times 0.1$ mm^3, baffle size: $5 \times 20 \times 0.01$ mm^3. Incident beam: 532 nm, 2 mm in diameter.

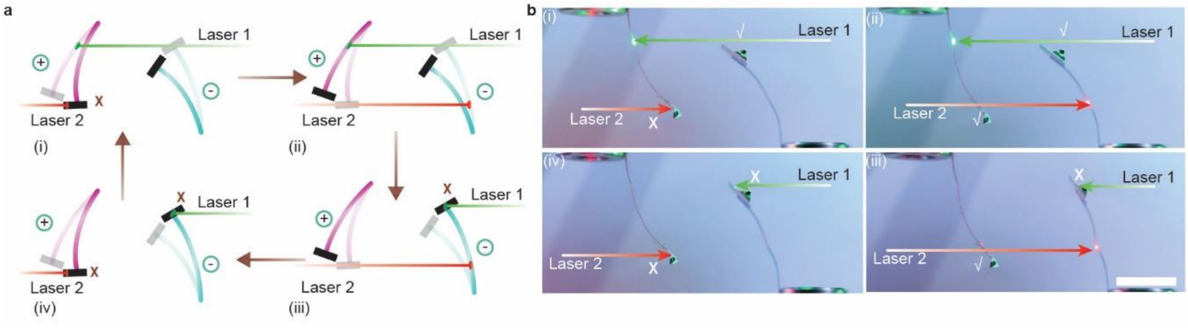

Supplementary Figure 6. Kinetics of coupled oscillators. (a) A schematic illustration detailing the deformation steps of two operators. (b) Corresponding images depict the shape-change of actuators within one oscillation cycle. Scale bar: 1 cm.

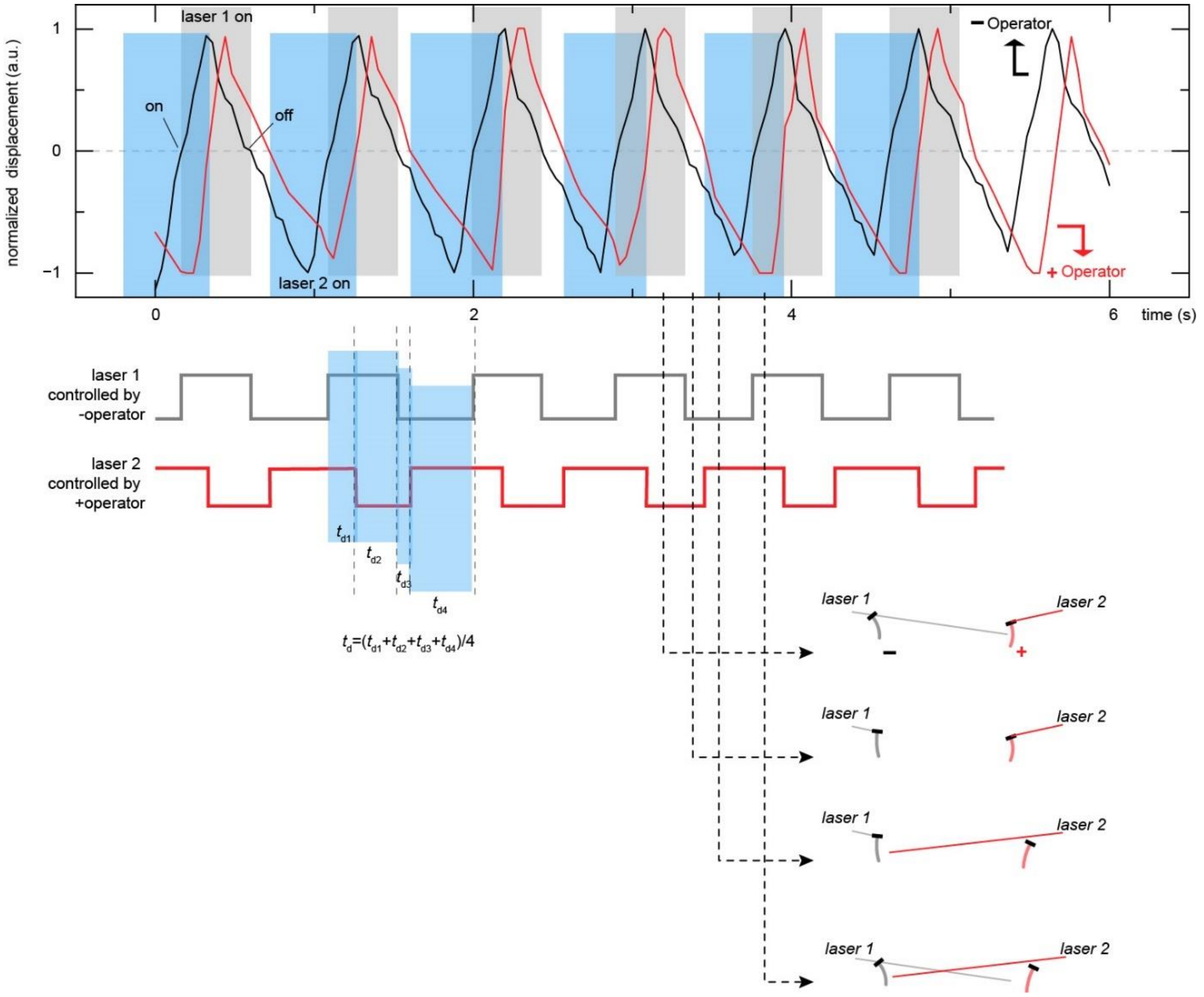

Supplementary Figure 7. Displacement kinetics explanation. In the experiment, the parameters are Laser 1: 180 mW, laser 2: 150 mW, l_- = 2.5 cm, l_+= 2.5 cm. t_d, is the time delay. t_{d1}: time between laser 1 ON and laser 2 OFF. t_{d2}: time between laser 2 ON and laser 1 ON. t_{d3}: time between laser 1 OFF and laser 2 ON. t_{d4}: time between laser 2 OFF and laser 1 OFF.

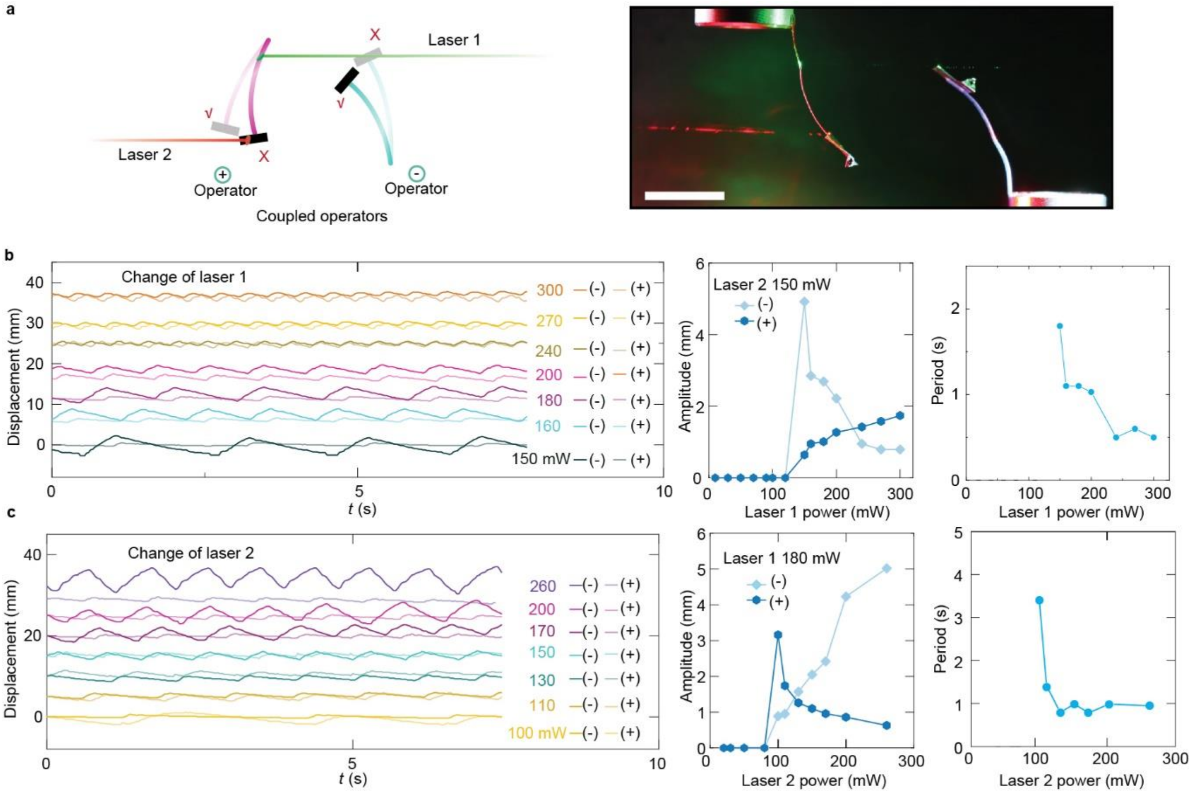

Supplementary Figure 8. Light power dependent behaviors in the coupled oscillators (a) Left: A schematic illustration depicts (-) and (+)operators coupled through two laser beams. Right: A photographic depiction showcases the optical setups of the coupled system. (b) Left: oscillation data recorded by varying the power of laser 1 while maintaining the constant power of laser 2. Right: the variation in amplitude and period of oscillation with changes in the power of laser 1. Laser 2: 150 mW, 635 nm, 3 mm spot size. (c) Left: oscillation data recorded by varying the power of laser 2 while maintaining the constant power of laser 1. Right: the variation in amplitude and period of oscillation with changes in laser 2 power. Laser 1: 180 mW, 532 nm, 2 mm spot size. Scale bar: 1 cm.

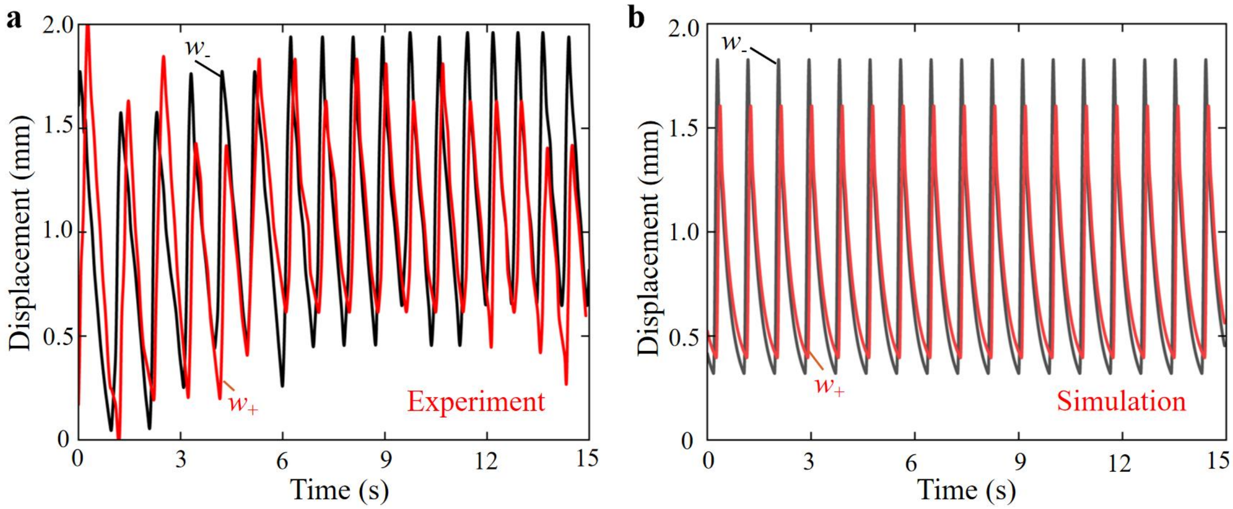

Supplementary Figure 9. Modelling fitting. (a) Experimental result oscillation data of the coupled oscillators. Displacement shows the change of tip positions of the two baffles. Laser (-): 130 mW, laser (+): 180 mW. (b) Theoretical prediction. In the simulation, we set l_{-} = 2.5 cm, l_{+}= 2.5 cm, w_{0-}= 4.25 mm, w_{0+}= 4.37 mm, $\bar{\beta}$ = 0.8, τ_{inertial} = 0.015 s, τ_{heat} = 0.3 s, P_{-} = 130 mW, P_{+} = 180 mW, λ_{-} = 0.0017 /mW, λ_{+} = 0.001 /mW.

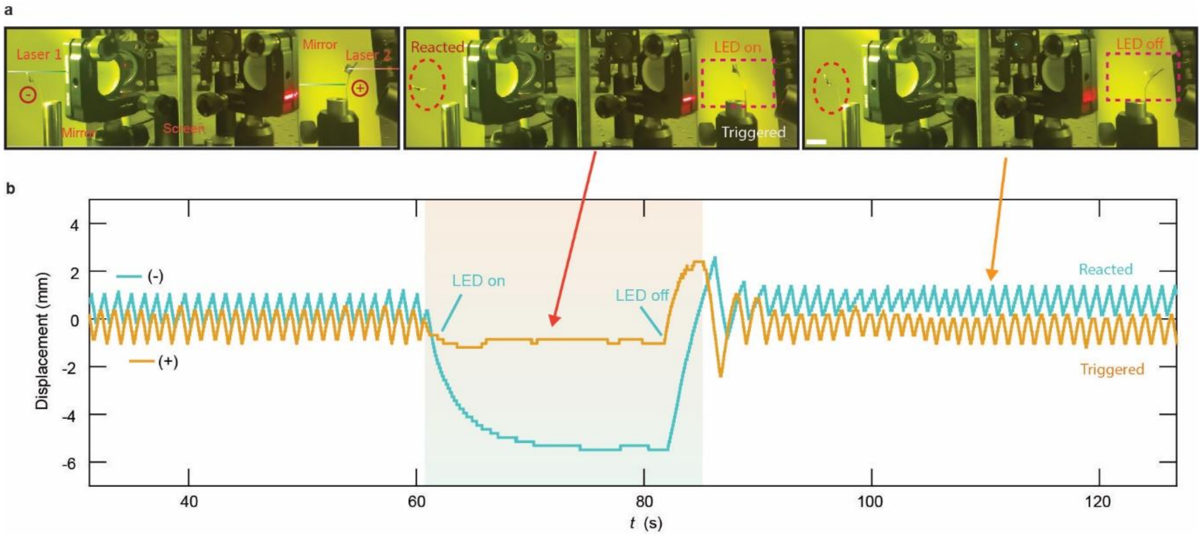

Supplementary Figure 10. Interdependency of two coupled operators. (a) Photographic demonstrating physically isolated operators at different oscillation phases. (b) Oscillation data upon manual interruption by illuminating LED light onto the (+)operator. The LED light (635 nm, 15 mW cm^{-2}) causes a cessation of vibration. The shadowed area indicates the duration of the light disturbance. Laser spot sizes are 2 mm (laser 1) and 3 mm (laser 2). Laser powers are 70 mW (laser 1) and 540 mW (laser 2). Wavelengths are 532 nm for laser 1 and 635 nm for laser 2. Scale bar: 1 cm.

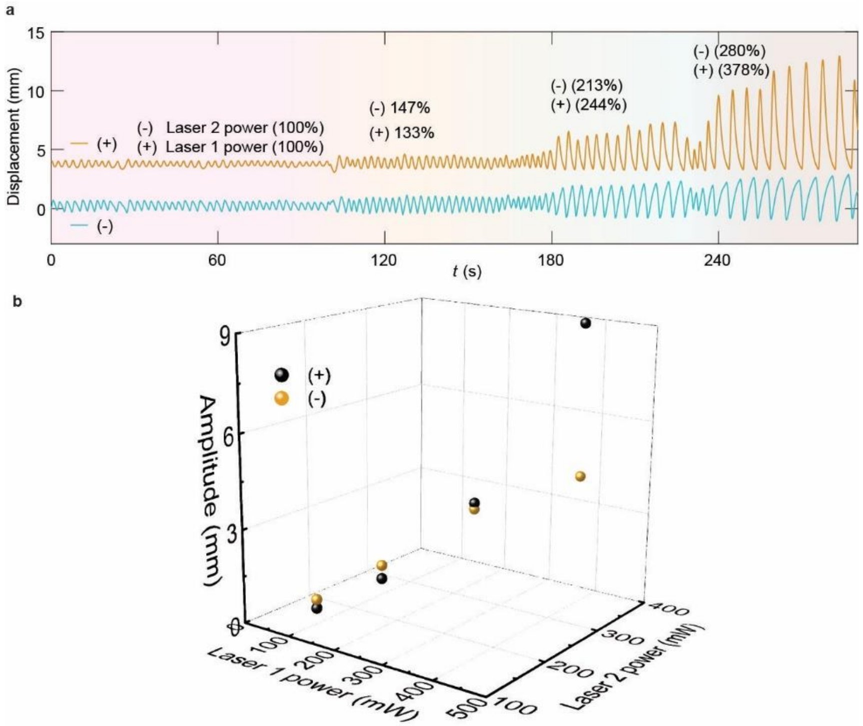

Supplementary Figure 11. Total power dependency in coupled oscillation. The powers of two laser beams are increased simultaneously. (a) Oscillation data by changing the power of both laser beams. (b) Corresponding amplitude variations in both operators. Two operators are physically isolated by using a screen board. The initial power of the beams is 150 mW (laser 1) and 90 mW (laser 2). The spot sizes are 2 mm (laser 1) and 3 mm (laser 2). Excitation wavelengths are 532 nm (laser 1) and 635 nm (laser 2).

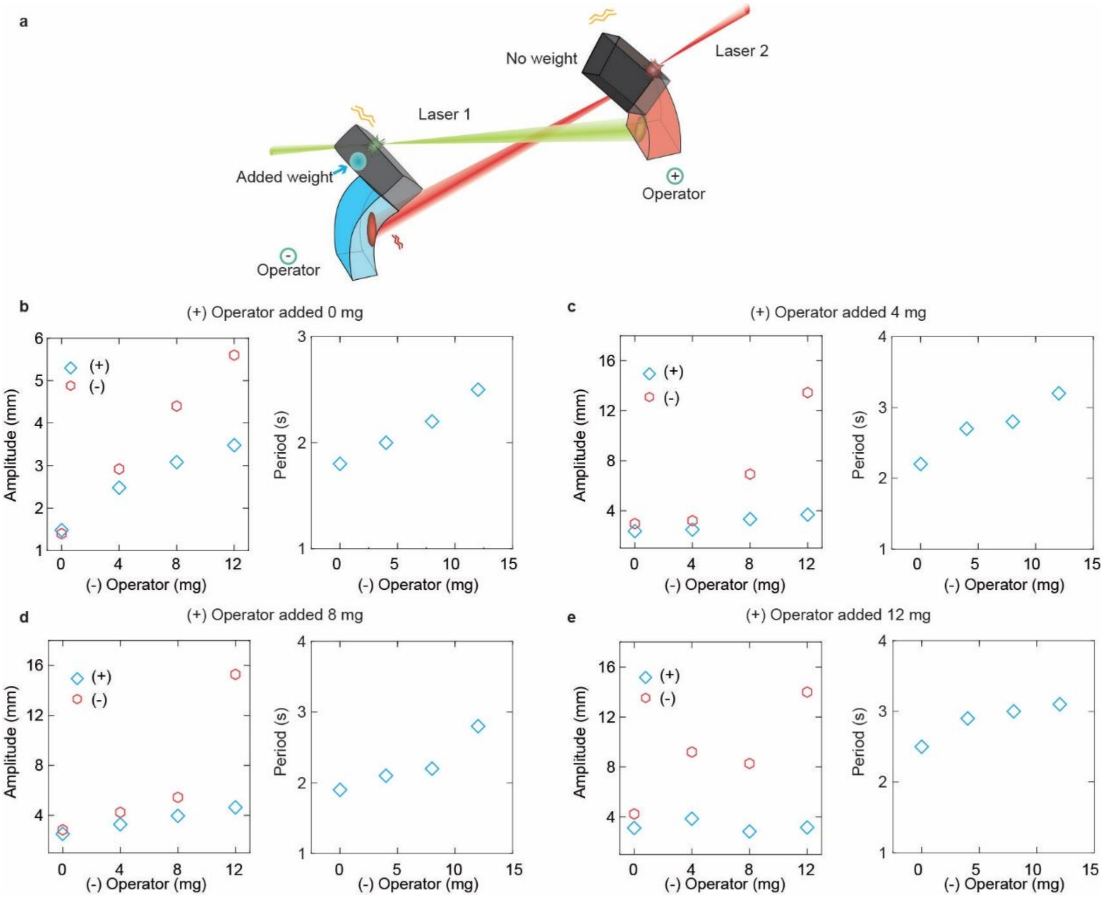

Supplementary Figure 12. Effect of added mass. (a) Schematic depiction of two coupled operators. Variation in amplitude and period by adding mass onto (-)operator, while keeping the added mass on (+)operator at 0 mg (b), 4 mg (c), 8 mg (d), 12 mg (e). Two operators are physically isolated by using a screen board. Light power is 220 mW for both lasers. Spot sizes are 2 mm for laser 1 and 3 mm for laser 2. Excitation wavelengths are 532 nm (laser 1) and 635 nm (laser 2).

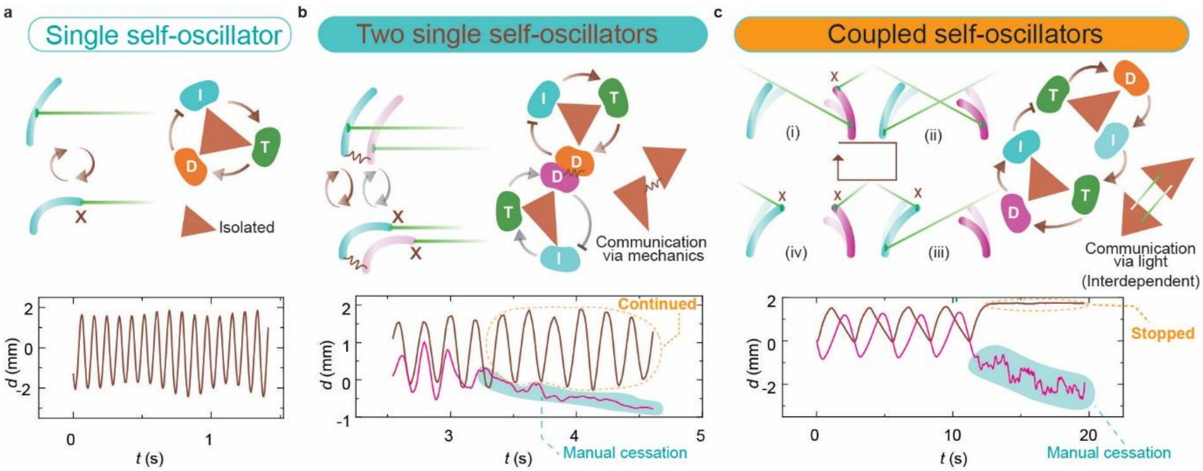

Supplementary Figure 13. The concepts of interaction in non-equilibrium matters. (a) The conventional single-piece self-oscillator is built upon a negative feedback loop. Bottom: oscillation data depicts an LCE-based self-oscillator based on the design shown in Fig.1d. (b) In a mechanically connected oscillating system, each oscillator is driven by a feedback loop on its own and interacts with others through physical interaction. Bottom: oscillation data depicts two LCE self-oscillators, in which manual cessation of one-unit unaffecting the oscillation dynamics of the other. (c) The interdependent self-oscillators are coupled through light beams. In this case, two units rely on each other to sustain the motion. Bottom: oscillation data showcases the displacement of two coupled oscillators, in which manual cessation of one-unit stops the motion of the other.

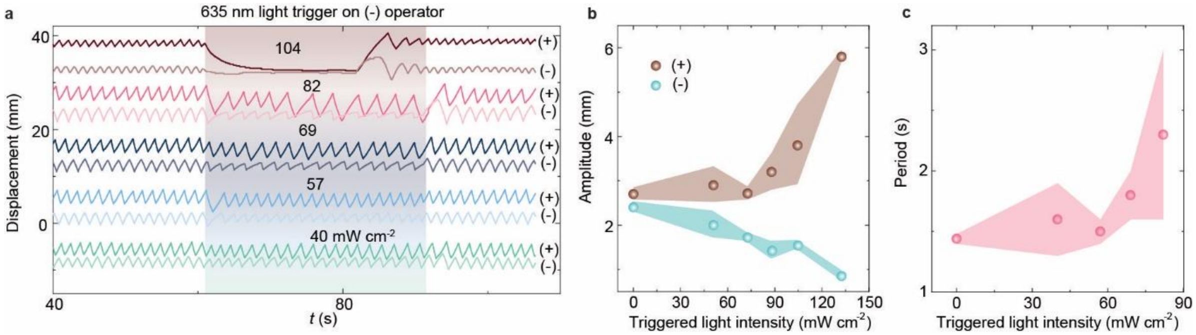

Supplementary Figure 14. Effect of additional illumination. (a) Oscillation data upon varying the intensity of external light interruption. External light: 635 nm, LED source, 40 to 104 mW cm^{-2}. The light trigger is imposed onto the (-)operator. (b) Amplitude variation of two oscillations with an increase of external light intensity for disturbance. (c) Period variation of the coupled operators by changing external light disturbance. Two operators are physically isolated by using a screen board. The power of the beams is 70 mW (laser 1) and 540 mW (laser 2). The spot sizes are 2 mm (laser 1) and 3 mm (laser 2). Excitation wavelengths are 532 nm (laser 1) and 635 nm (laser 2). Error bars represent s.d. for n = 3 measurements. The same sample was measured repeatedly.

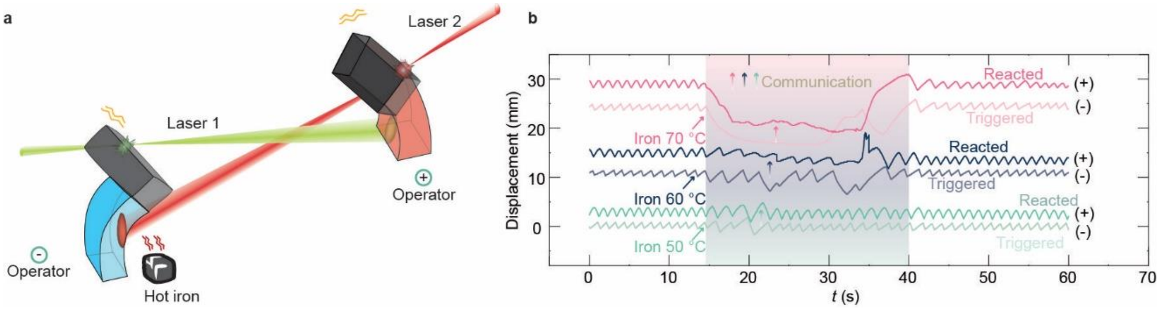

Supplementary Figure 15. Effect of heat disturbance. (a) Schematic diagram of the temperature interference in coupled operators. (b) Oscillation data upon different heat interferences. A hot iron (1.5 × 1.5 × 1.5 cm^3) was heated (50 to 70 ºC) and placed 2 cm below the (-)operator. Two operators are physically isolated by using a screen board. Laser 1: 532 nm, 70 mW. Laser 2: 635 nm, 540 mW. The spot sizes are 2 mm (laser 1) and 3 mm (laser 2).

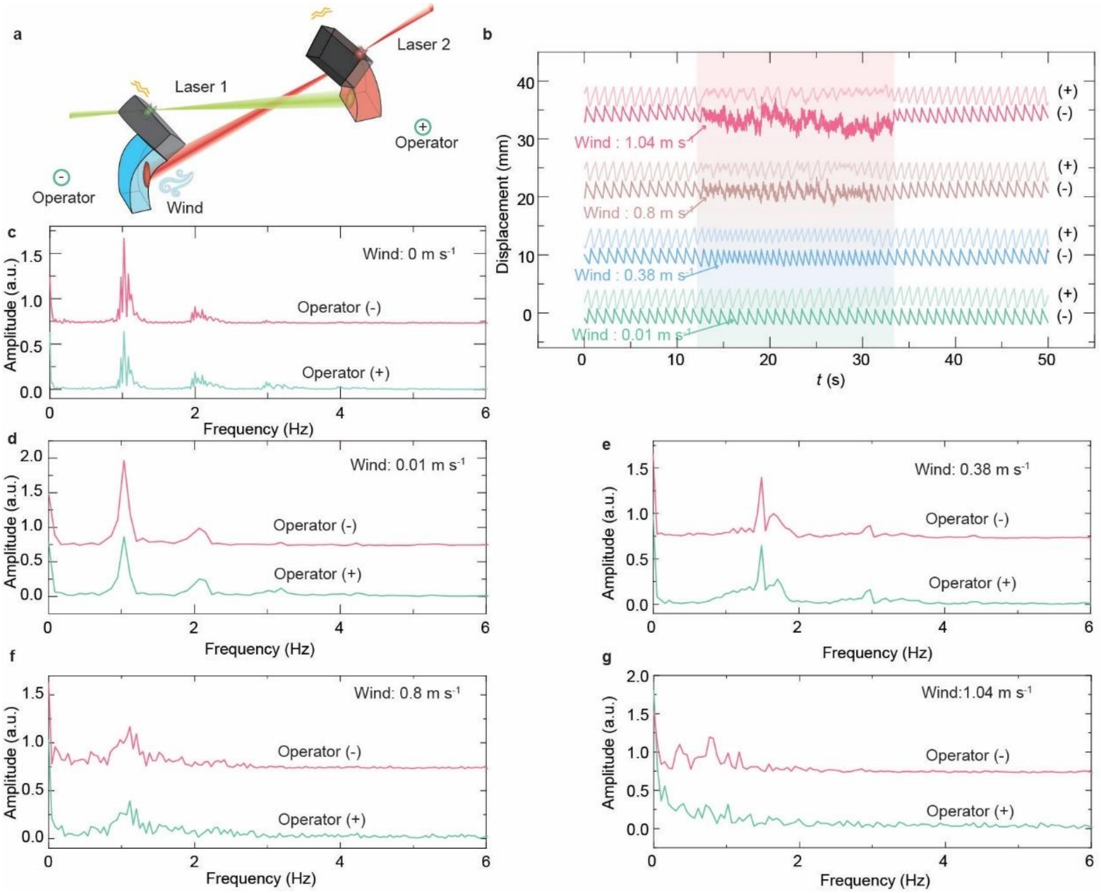

Supplementary Figure 16. Effect of wind disturbances. (a) Schematic diagram of the wind interference in the coupled operators. (b) Oscillation data of two operators, while the (-)operator is affected by wind flow with different velocities. Fourier transform spectra of oscillations at static air condition (c), upon 0.01 m s^{-1} (d), 0.38 m s^{-1} (e), 0.8 m s^{-1} (f) and 1.04 m s^{-1} (g) wind interferences. Fourier transform at the frequency domain is calculated from (b). Two operators are physically isolated by using a screen board. Laser 1: 532 nm, 70 mW. Laser 2: 635 nm, 540 mW. The spot sizes are 2 mm (laser 1) and 3 mm (laser 2).

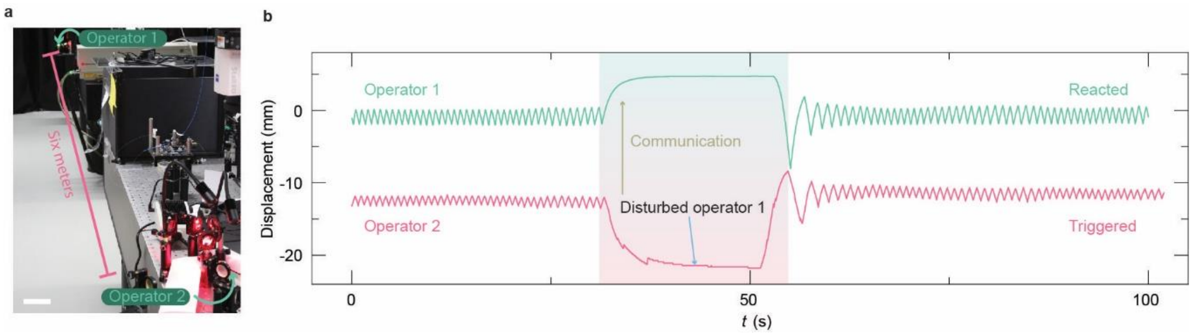

Supplementary Figure 17. Long distance signal transmission. (a) Photograph of light communication between two operators set on two optical tables separated six meters away from each other. The arrows point towards the positions of the operator. (b) Oscillation data of long distance coupled operators. One is ceased by manual stoppage. The shaded area represents the duration of the mechanical interference. Laser 1: 532 nm, 300 mW. Laser 2: 635 nm, 460 mW. The spot sizes are 2 mm (laser 1) and 3 mm (laser 2). Scale bar: 10 cm.

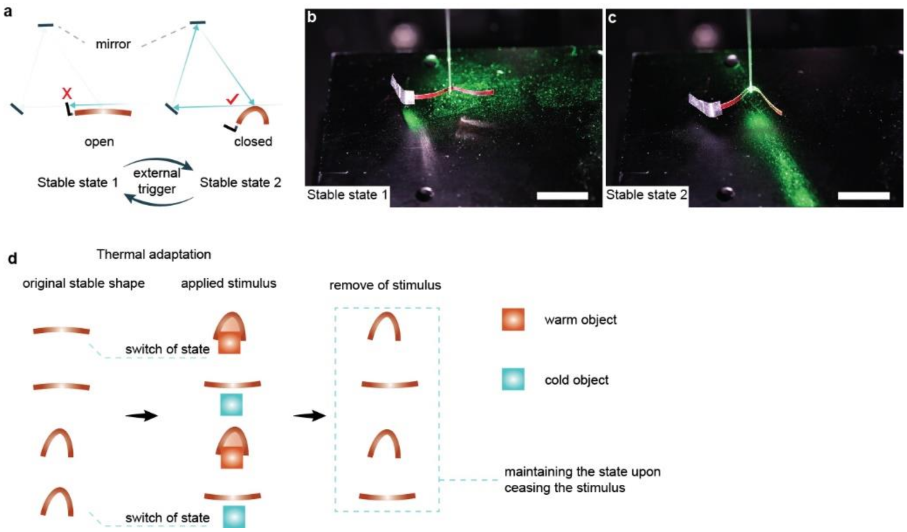

Supplementary Figure 18. Bi-stability enabled by positive feedback. (a) Optical design of a positive feedback loop consisting of one positive operator and a single light beam. Photos of the operator in the (b) open state and (c) closed state. (d) Four scenarios illustrating thermal adaptation, showing shape changes in response to heat or cold stimuli, and the capability of maintaining the state after ceasing the stimulus. Scale bar is 1 cm.

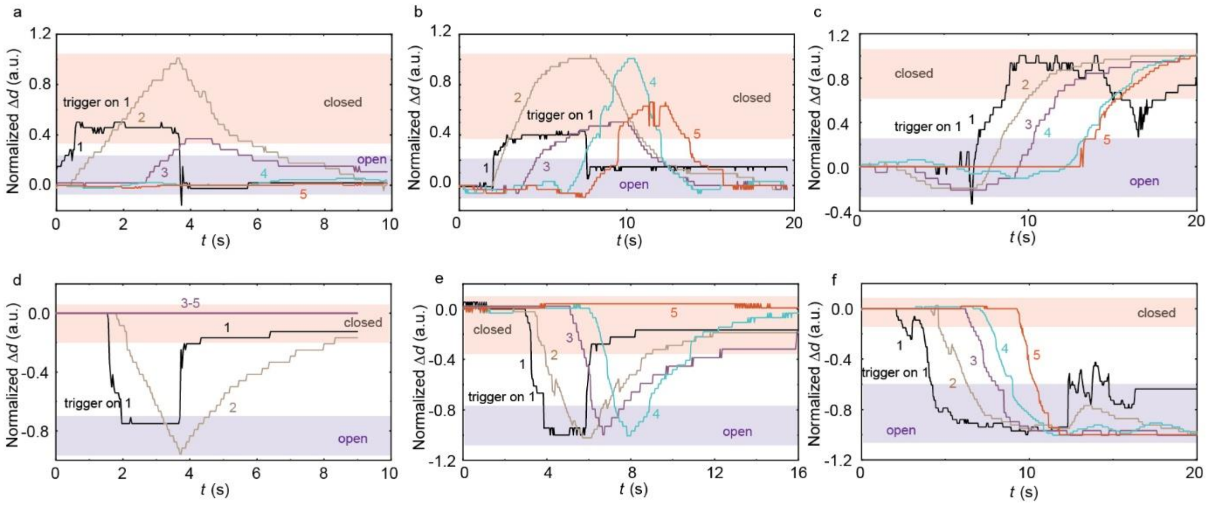

Supplementary Figure 19. Displacement data during cascading transition. (a-c) Initially, all operators are in the open state. The change in baffle position (Δ*d*) for each operator is recorded by applying a mechanical trigger on operator 1 for (a) 2.5 s, (b) 5 s, and (c) 13 s. (d-f) Initially, all operators are in the closed state. The change in baffle position (Δ*d*) for each operator is recorded by applying a mechanical trigger on operator 1 for (a) 2 s, (b) 4 s, and (c) 7 s.

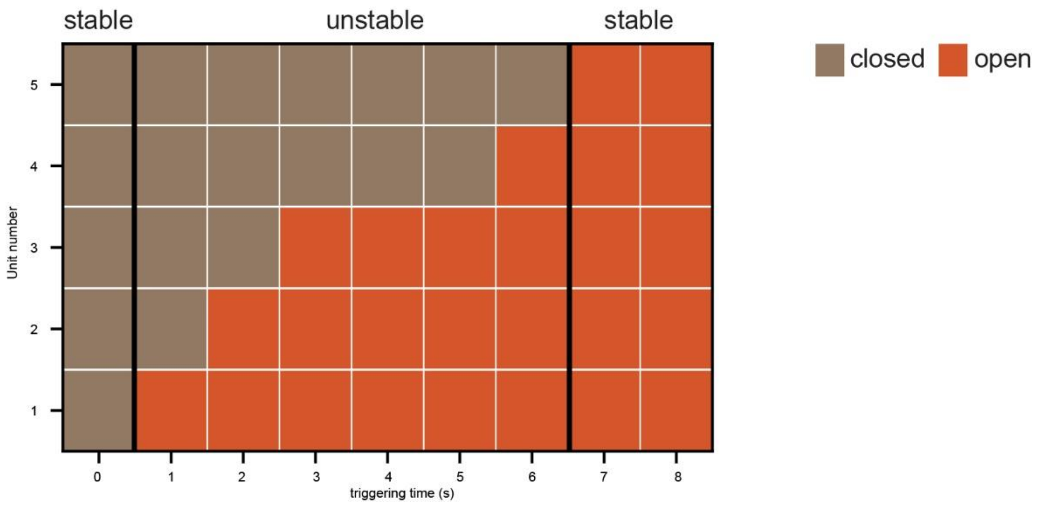

Supplementary Figure 20. Cascading events in state transition. The network consists of five positive operators in a closed loop, initially all in the closed state. The state changes of each operator are recorded as varying trigger intervals are applied to the first operator.

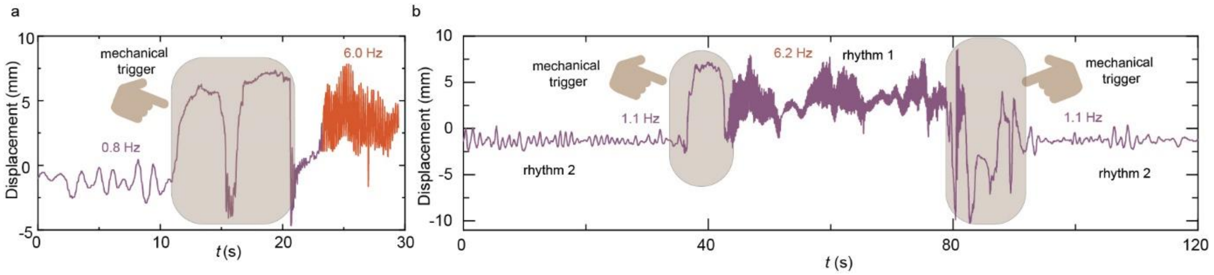

Supplementary Figure 21. Change in rhythm. (a) Oscillation data showing the transition from low-frequency to high-frequency oscillation triggered by a mechanical stimulus. (b) Oscillation data illustrating transitions from low to high frequency and back to low frequency, induced by different mechanical triggers.

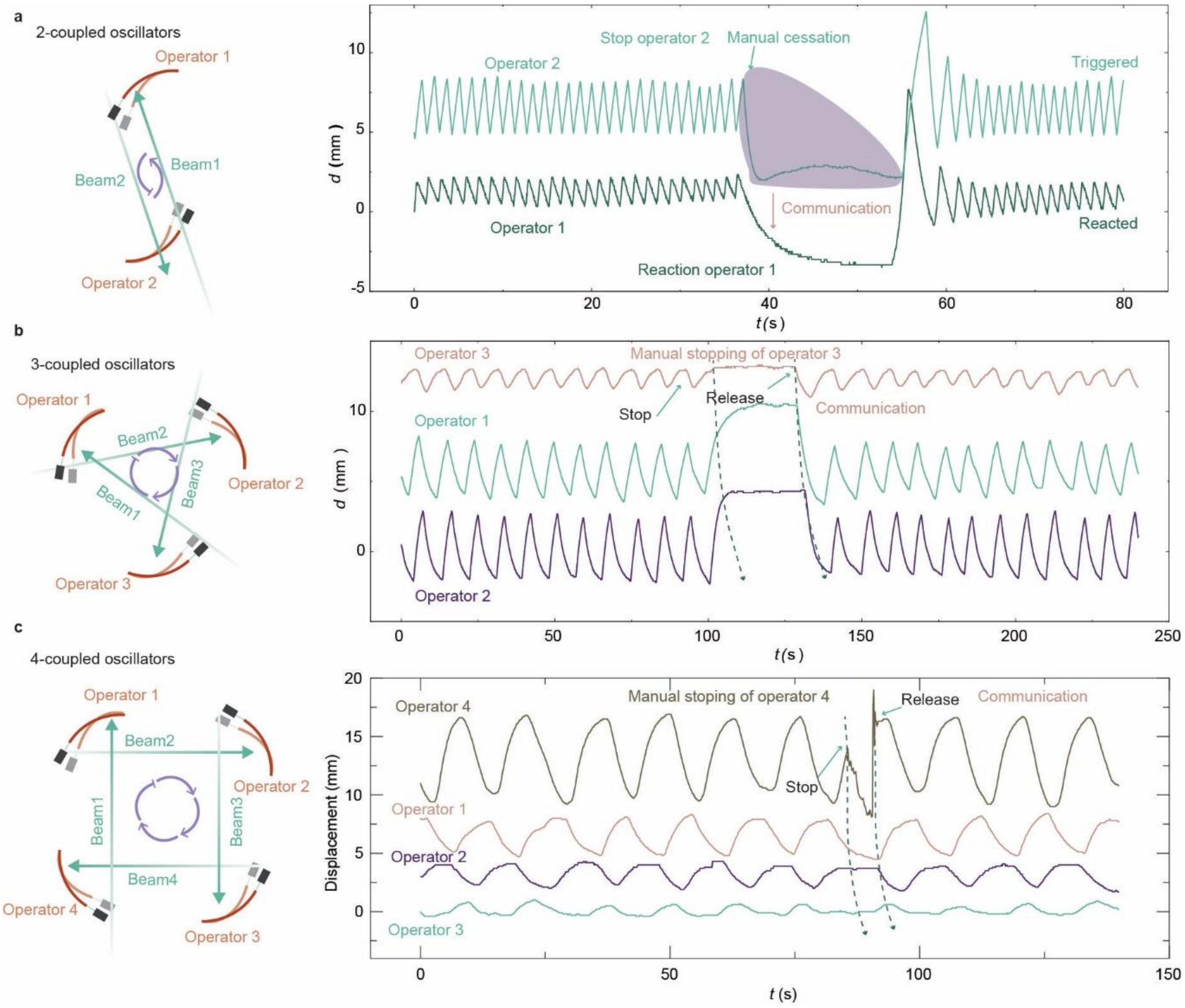

Supplementary Figure 22. Light communicative network. (a) Schematic diagram depicting the mechanism of a self-oscillating system consisting of two units, accompanied by oscillation data of the operators. The purple area indicates the duration of the manual stoppage. Laser 1: 532 nm, 70 mW. Laser 2: 635 nm, 540 mW. The spot sizes are 2 mm (laser 1) and 3 mm (laser 2). (b) Schematic diagram of a system composed of three units, along with corresponding oscillation data. All laser: 532 nm, 320 mW. Laser spot sizes: 2 mm. (c) Schematic diagram illustrating a self-oscillating system composed of four units, accompanied by oscillation data. All laser: 532 nm, 320 mW. Laser spot sizes: 2 mm. LCE actuator dimensions in all cases: $24 \times 2 \times 0.1$ mm^3. Baffle dimension: $5 \times 20 \times 0.01$ mm^2.

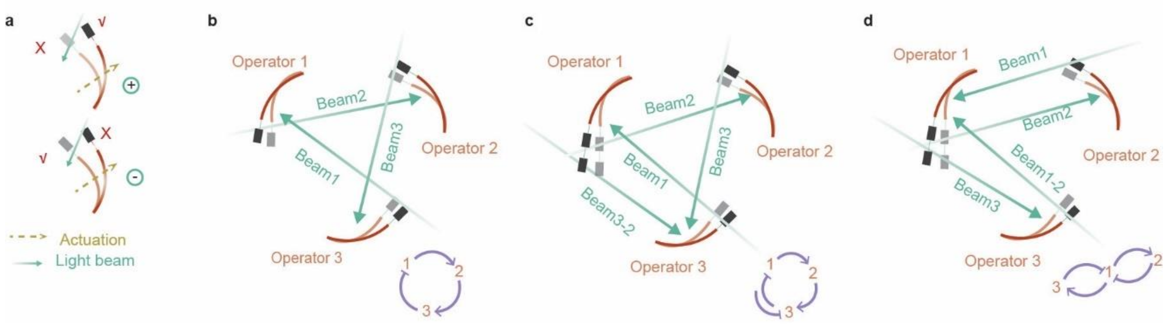

Supplementary Figure 23. Designs of bio-inspired light communicative network. (a) The schematic diagram illustrates the mechanism of a single operator. (b) The three-component negative feedback loop represents a basic form of a bio-oscillator, where feedback mechanisms regulate the activity of components within the system. This is the most typical configuration often resulting in rhythmic oscillations based on the interplay of activating and inhibiting signals. (c) The incoherent feedforward loop introduces an additional negative feedback mechanism to enhance the robustness of the oscillatory behavior. It usually makes the system more resilient to external disturbances. (d) In the configuration with two negative feedback loops acting on the same operator, chaos may ensue. This scenario may lead to highly unpredictable and irregular oscillations, characterized by complex dynamics and a lack of stable patterns. The designs are inspired by Ref [1].

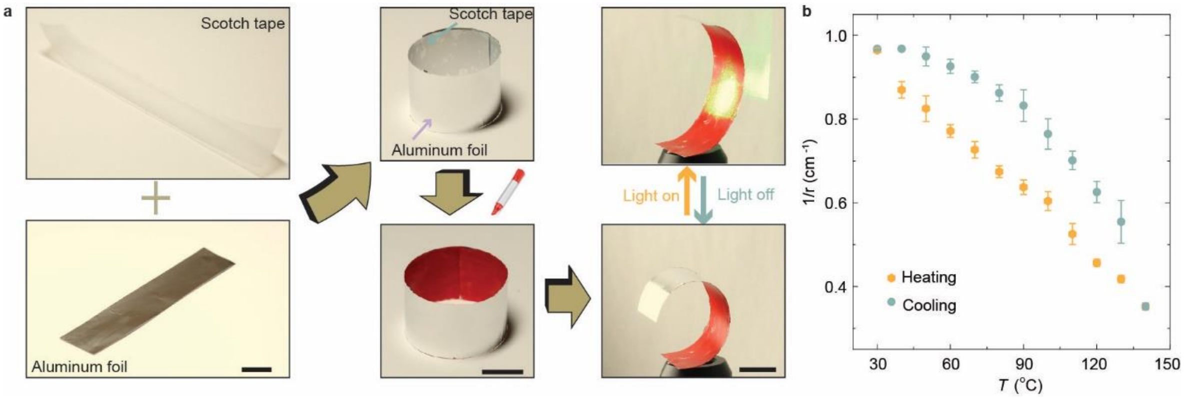

Supplementary Figure 24. Responsive behaviors in aluminum foil-scotch tap bilayer. (a) Preparation steps. A thermally responsive bilayer actuator is comprised of two kinds of daily material, *i.e.*, scotch tape (50 µm thick) and aluminum foil (10 µm thick). After sticking two films together and painting with a red colored pen on the scotch tape, the bilayer is annealed on a hotplate at 140 ºC. After annealing, the bilayer exhibits reversible deformation based on the photothermal effect. Light illumination: 460 nm, 50 mW cm^{-2}. (b) Curvature (1/r) variation upon temperature change. All scale bars are 1 cm.

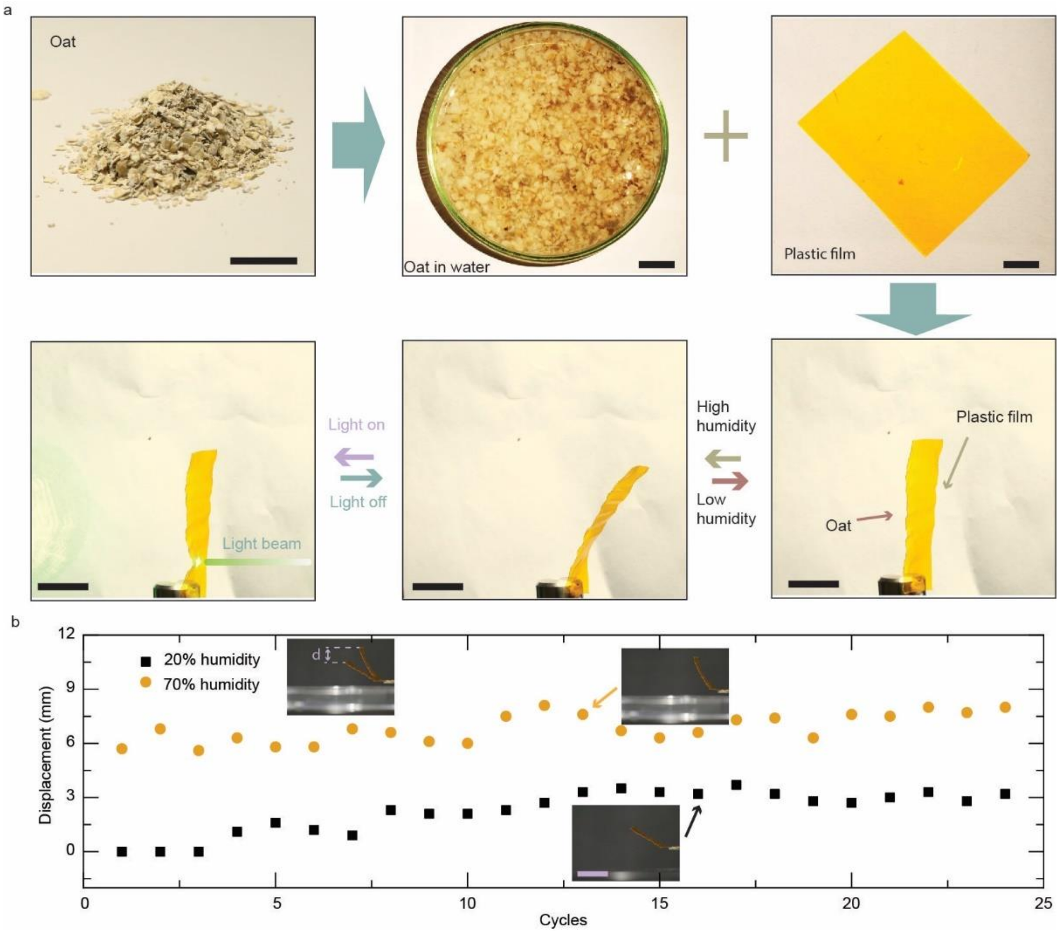

Supplementary Figure 25. Responsive behaviors in the oat-plastic bilayer. (a) The depicted process illustrates the preparation of a humidity-responsive film derived from common oats. The procedure involves dissolving oatmeal flakes into water, boiling the water, and drop casting oat solution onto a colored plastic film as a passive layer. After water evaporation, the bilayer exhibits reversible deformation upon a change in ambient humidity. A light excitation can also cause desorption of water content from the oat layer, inducing reversible photo-heat-humidity triggered deformation. Light: 532 nm laser, 90 mW, spot size, 2 mm. (b) The cyclic stability of the humidity-responsive film under multiple cycles of humidity switching between 20% and 70%. The inset defines displacement and shows photos of the film bending at different humidity levels. All scale bars are 1 cm.

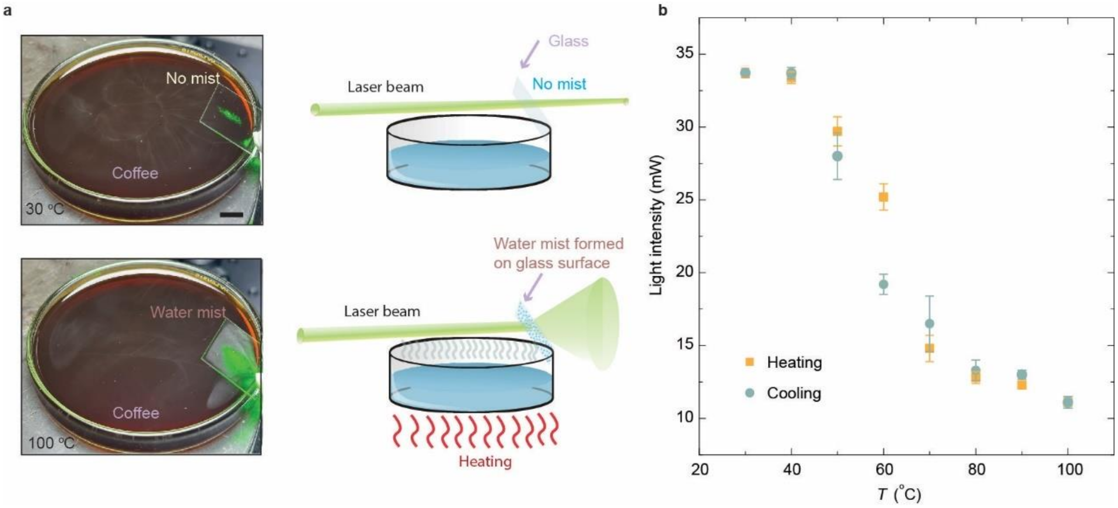

Supplementary Figure 26. Responsive behaviors in a foggy glass. (a) Photographs and schematic diagrams depicting the fogging formation on the cover glass slide on top of the coffee container. The moisture from the heated coffee creates a foggy layer that drops the transparency of the cover glass. (b) Light transmission through the cover glass was measured at different coffee temperatures. Light: 532 nm laser beam, input power 33 mW. Error bars represent s.d. for n = 3 measurements. The same sample was measured repeatedly. Scale bar: 1 cm.

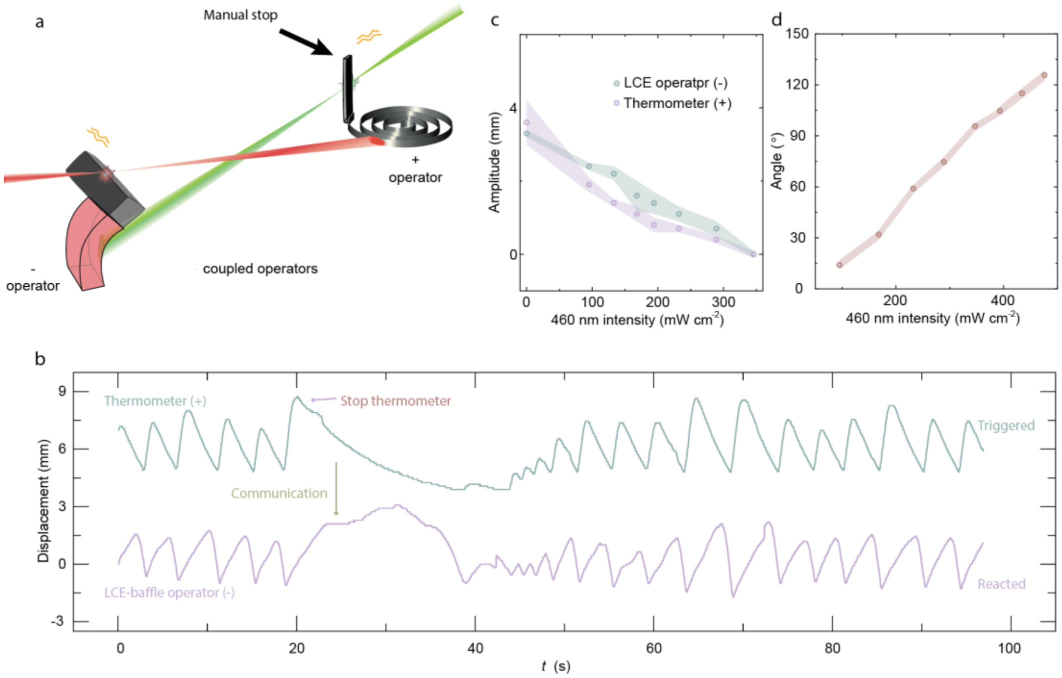

Supplementary Figure 27. Signal transmission between LCE actuator and thermometer spring. (a) The schematic diagram illustrates the mechanism of the light communicative system based on the coupling between an LCE-baffle and a thermometer-baffle. The side of the thermometer spring is blackened with a pen to enhance photo-heat absorption. (b) Oscillation data of the coupled system, in which a manual halting at the thermometer and subsequent synchronization between two-oscillator motions are showcased. Laser 1: 532 nm, 930 mW, 3 mm spot size. Laser 2: 532 nm, 44 mW, and 2 mm spot size. (c) Variation of oscillation amplitude upon external light disturbance on the thermometer. Light disturbance: LED source, 460 nm, 0 to 350 mW cm^{-2}. (d) The graph presents the photothermal responsive characteristics of the thermometer spring. LCE actuator dimensions: 24 × 2 × 0.1 mm^3. Baffle dimension: 5 × 20 × 0.01 mm^2. Error bars represent s.d. for n = 3 measurements. The same sample was measured repeatedly.

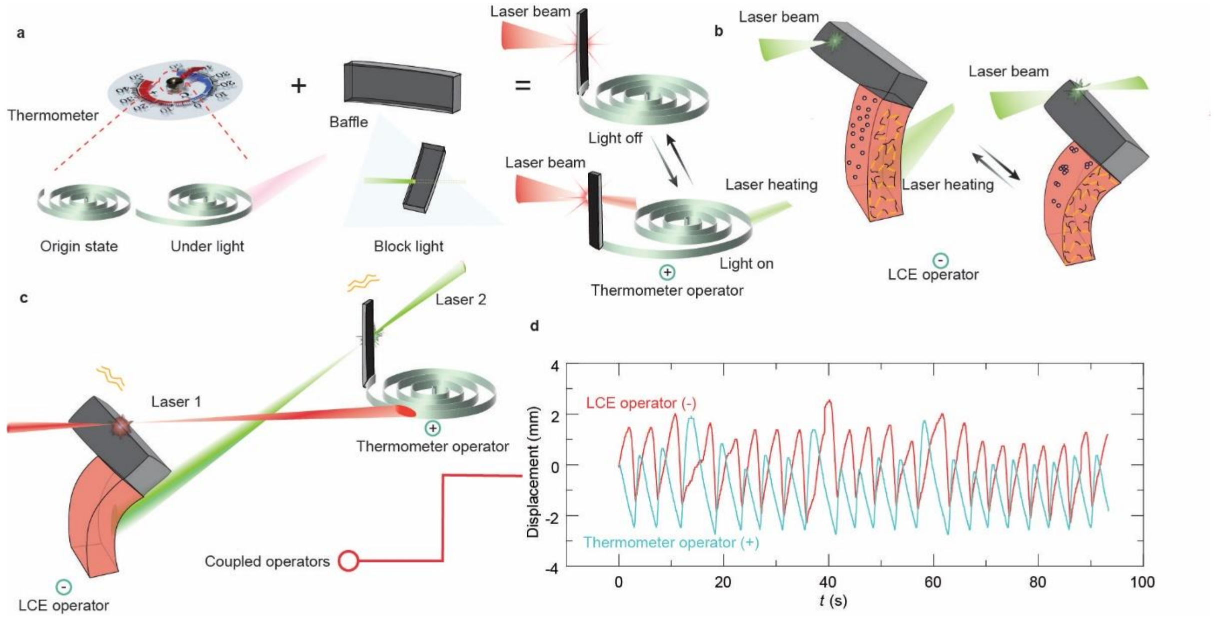

Supplementary Figure 28. Coupling between LCE and a thermometer spring. (a) Schematic drawing showing the essential components of the thermometer-baffle operator. The thermometer metal spring undergoes bending upon exposure to light, an aluminum foil sheet acts as a barrier preventing light transmission. (b) A schematic diagram demonstrates the bending behavior of the LCE-baffle (-)operator when subjected to light irradiation. (c) The schematic diagram depicts the coupling between the thermometer and the LCE actuator. (d) Oscillation data of the coupled system. Laser 1 power: 930 mW, Laser 2 power: 44 mW, all beams are 532 nm. The size of the light spot for laser 1 is 3 mm, and for laser 2 is 2 mm. LCE sample dimensions: $24 \times 2 \times 0.1$ mm^3, baffle size: $5 \times 20 \times 0.01$ mm^3.

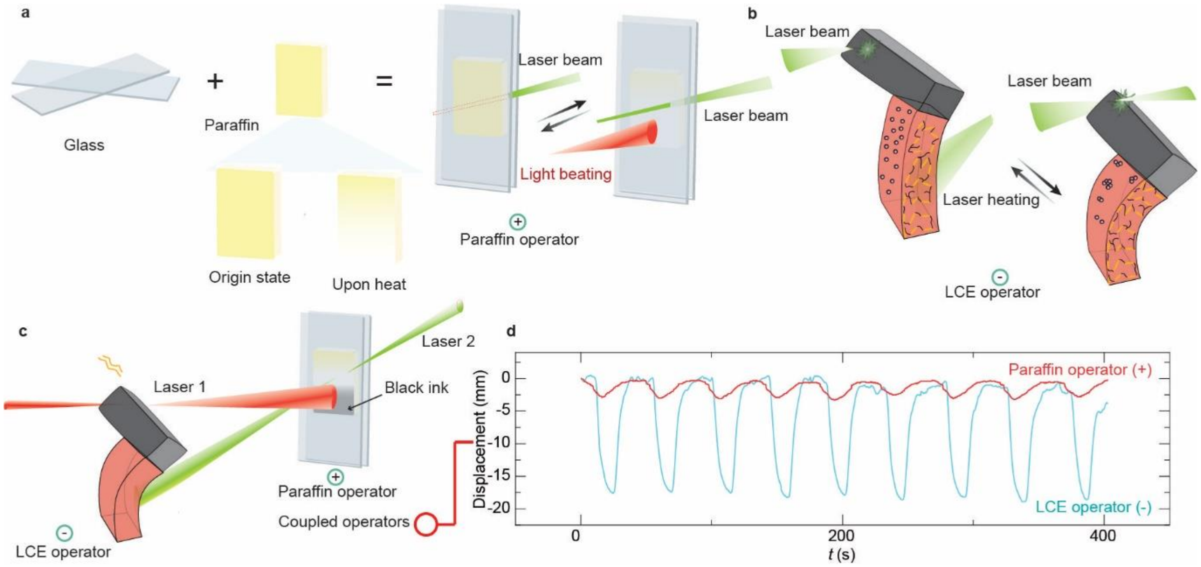

Supplementary Figure 29. Coupling between LCE and a paraffin plate. (a) The assembly includes all essential components for operator systems. Glass substrates are used to form a cell to store the paraffin. Paraffin becomes transparent to light when heated. (b) A schematic diagram illustrates the bending behavior of the LCE-baffle (-)operator when subjected to light irradiation. (c) The schematic diagram depicts the coupling between a paraffin plate and an LCE-baffle operator. (d) Oscillation data of the coupled system. Laser 1 power: 1440 mW, Laser 2 power: 80 mW, all beams are 532 nm. The spot size for laser 1 is 2 mm, and 1.2 mm for laser 2. LCE sample dimensions: $24 \times 2 \times 0.1$ mm^3, baffle size: $5 \times 20 \times 0.01$ mm^3.

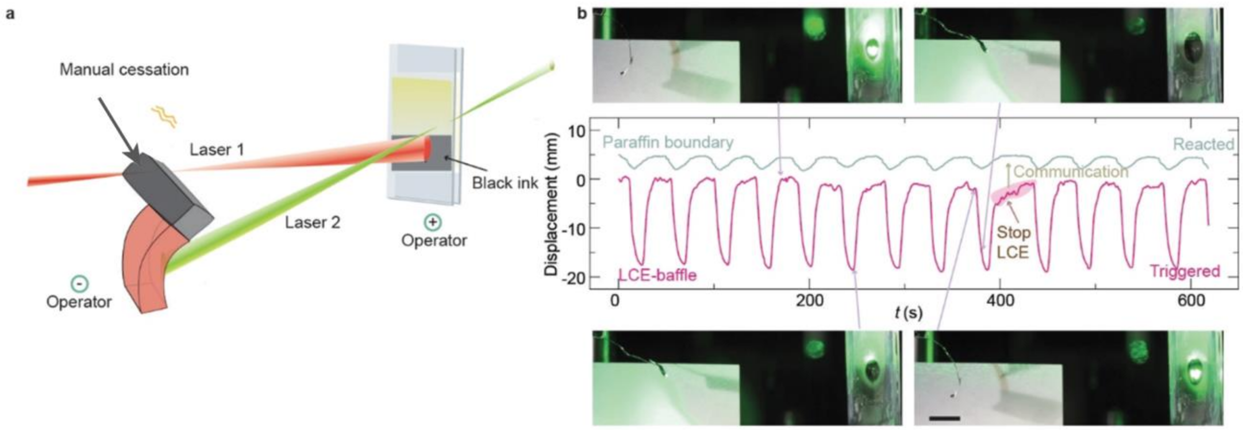

Supplementary Figure 30. Signal transmission between LCE actuator and paraffin. (a) Schematic diagram illustrating the mechanism of the light communicative system based on the coupling between an LCE-baffle and a glass cell infiltrated with paraffin. The lower section of the paraffin is blackened with a pen to enhance photo-heat absorption. (b) Oscillation data of the coupled system. Insets are photographs of the bending LCE operator and paraffin at different oscillation phases. Laser 1: 532 nm, 1440 mW, 2 mm spot size. Laser 2: 532 nm, 80 mW, and 1.2 mm spot size. LCE actuator dimensions: $24 \times 2 \times 0.1$ mm^3. Baffle dimension: $5 \times 20 \times 0.01$ mm^2. Scale bar: 1 cm.

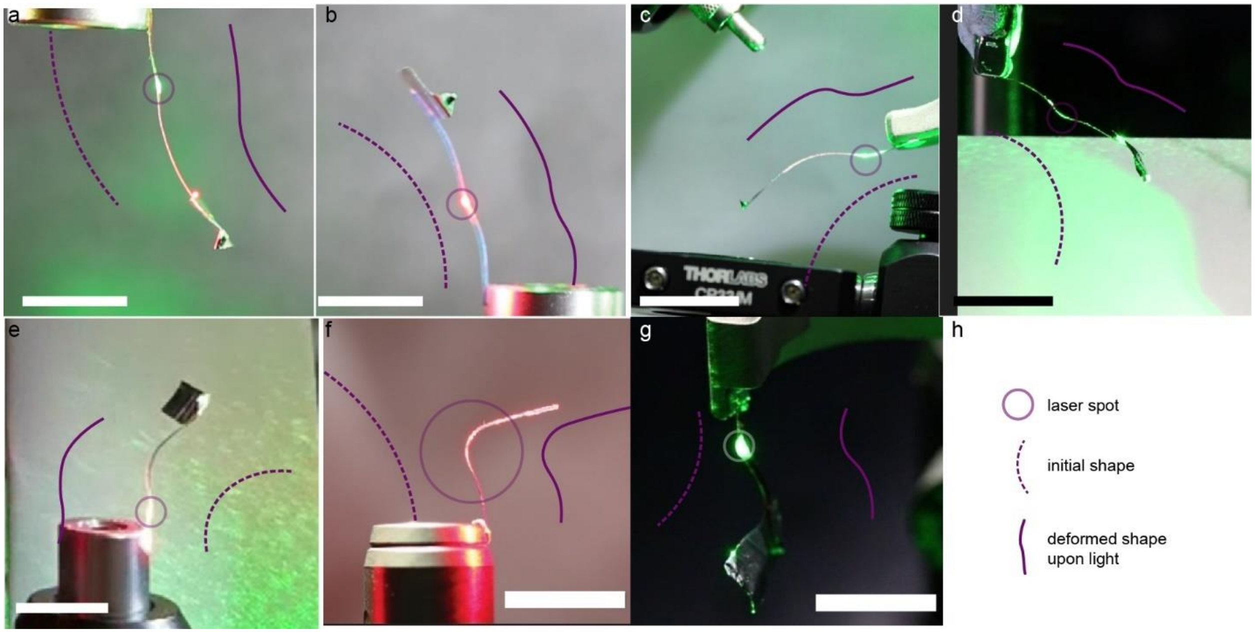

Supplementary Figure 31. Photographs of the shape-morphing for soft LCE deformation. The images are captured during the experiments illustrated in (a, b) Figure 2c, (c) Figure 3h, (d) Figure 5g, (e) Figure 3b, (f) Supplementary Fig. 3b, (g) Figure 5e. (h) Indication of symbol.

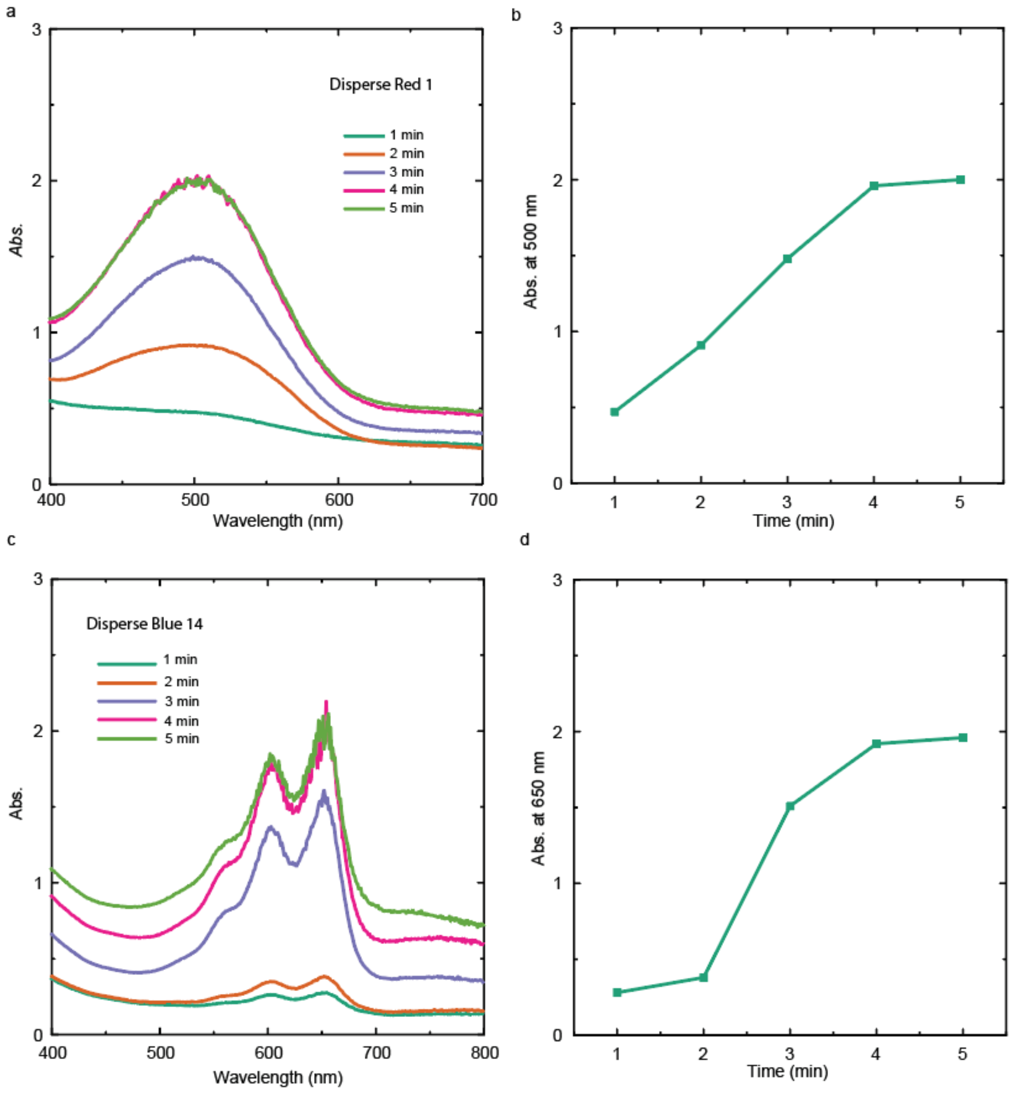

Supplementary Figure 32. Enhanced absorption during dying. (a) UV-vis spectra after thermal diffusion of Dispersed Red 1 and (b) the change of absorbance at the excitation wavelength (532 nm). (c) UV-vis spectra after thermal diffusion of Dispersed Blue 14 and (d) the change of absorbance at the excitation wavelength (635 nm). Sample thickness: 0.1 mm. Thermal diffusion is processed at 100 °C.

2. Supplementary Note

Theoretical considerations are summarized as follows: In Section 2.1, dynamic equations for the single oscillator coupled with the photothermally responsive LCE model are derived. Numerical solutions of these equations reveal a Hopf bifurcation between static and self-oscillation states, yielding the time history of the single oscillator. Amplitude and period variations for different laser powers are determined based on this time history. In Section 2.2, governing equations for two coupled oscillators are derived, emphasizing the time delay mechanism using the time histories of both oscillators. The impact of light powers on amplitude and period is examined. Section 2.3 compares the differences between the single self-oscillation mode and the coupled mode. It also examines the influence of delay distance on oscillation behavior. Section 2.4 discusses the potential use of coupled oscillators for remote robotic control and electrical signal transmission.

2.1 Single oscillator

Dynamics of the single oscillator. Figure 33 illustrates the single oscillator, consisting of an LCE beam, a baffle, a laser beam, and a mirror. Initially, the baffle allows the propagation of light, and the laser beam induces bending in the LCE through photothermal actuation (Figure 33a). Consequently, due to LCE deformation, the baffle impedes the light beam (Figure 33b). Subsequently, the light-induced bending rebounds, causing the LCE cantilever to unbend, thereby allowing the resumption of light propagation and initiating a new cycle. During the vibration, the baffle is subjected to the bending force F_b of the LCE beam, and the damping force F_d (Figure 33d), therefore the governing equation for its vibration is written as

$$m\frac{d^2 w(t)}{dt^2} = F_d + F_b, \quad (1)$$

where m is the mass of the system, w(t) is the end deflection of the LCE cantilever. For simplicity, the damping force is assumed to be proportional to the velocity of the baffle, *i.e.*,

$$F_d = -\beta\frac{dw(t)}{dt}, \quad (2)$$

in which, β is damping coefficient. For simplicity, the bending force of the LCE beam is assumed to be proportional to the elastic bending deformation, i.e.

$$F_b = -\frac{3\Pi}{l^3} w_e(t), \quad (3)$$

in which, Π is bending stiffness, l is the length of the LCE cantilever. As shown in Figure 33c, the elastic bending deformation $w_e(t)$ depends on both the light-driven bending deflection $w_L(t)$ and current bending deflection $w(t)$ (i.e. total bending deflection), which is calculated as

$$w_e(t) = w(t) - w_L(t). \tag{4}$$

For simplicity, the light-driven bending $w_L(t)$ is assumed to be proportional to the temperature difference $T_{dif}(t)$ between the temperature $T_{em}(t)$ at the illuminated position and the ambient temperature T_{am}, i.e.,

$$w_L = A[T_{em}(t) - T_{am}], \tag{5}$$

in which, A is the light-driven bending coefficient, depending on the detail of the system.

Inserting Eqs. (2)-(5), the governing equation (1) can be rewritten as

$$m\frac{d^2 w(t)}{d t^2} = -\beta\frac{d\,w(t)}{d\,t} + \frac{3\Pi}{l^3}[AT_{dif}(t) - w(t)]. \tag{6}$$

In Eq. (6), the temperature T(t) depends on the light illumination and is process-related due to the transition between cut-on state and cut-off state of the baffle. The self-oscillation originates from the temperature variation and movement of the baffle.

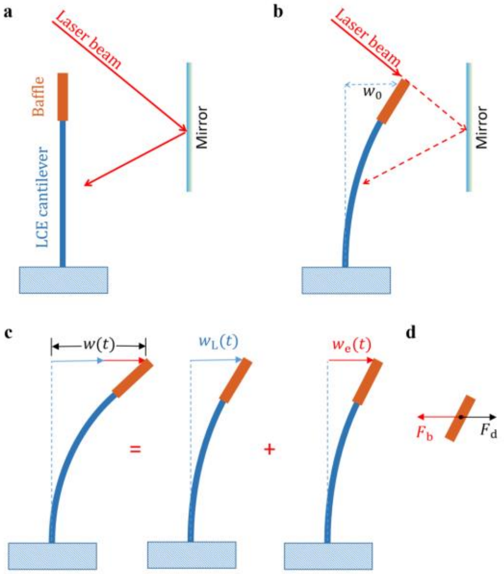

Figure 33. Schematics of a feedback self-oscillator. The oscillator is composed of an LCE cantilever and a baffle. (a) The laser beam induces bending in the LCE. (b) The baffle impedes the light beam upon bending. (c) The total bending deflection $w(t)$ equals to the sum of elastic bending deformation $w_e(t)$, and the light-driven bending deflection $w_L(t)$. (d) The directions of bending force F_b of the LCE beam, and the damping force F_d.

Model of a photothermally-responsive LCE beam. To obtain the temperature $T_{\mathrm{em}}(t)$ in Eq. (6), a photothermally-responsively LCE model is adopted. The LCE cantilever converts light energy into heat. Under light illumination, the temperature difference T_{dif} is determined by the heat flux from the light and the heat transfer into the ambient, *i.e.*

$$\rho_c \dot{T}_{\mathrm{em}} = q_l - q_{am}, \tag{7}$$

where ρ_c is the specific heat capacity, q_l is the heat absorbed by the LCE cantilever from light per second, which is assumed to be proportional to the light power P of the light beam, i.e.,

$$q_l = \eta P, \tag{8}$$

where η is the energy absorption coefficient. The LCE cantilever also exchanges heat with the environment, and the heat flux is assumed to be linear to the temperature difference T_{dif} between the LCE cantilever and the environment, *i.e.*,

$$q_{\mathrm{am}} = kT_{\mathrm{dif}}, \tag{9}$$

where k is the heat transfer coefficient.

Inserting Eqs. (8) and (9) into (7) the temperature difference T_{dif} is governed by

$$\dot{T}_{\mathrm{dif}} = \frac{\eta P - kT_{\mathrm{dif}}}{\rho_c}. \tag{10}$$

Solving Eq. (10), in illuminated state, *i.e.* $w < w_0$ or $P \neq 0$, the temperature difference T_{dif} follows the law:

$$T_{\mathrm{dif}} = T_{\mathrm{Limit}}\left(1 - e^{-t/\tau_{\mathrm{heat}}}\right), \tag{11}$$

while in non-illuminated state, i.e. $w > w_0$ or $P = 0$, the temperature difference T_{dif} follows the law:

$$T_{\mathrm{dif}} = T_{\mathrm{Limit}} e^{-t/\tau_{\mathrm{heat}}}, \tag{12}$$

where, $T_{\mathrm{Limit}} = \eta P/k$ represents the limit temperature difference of photothermally-responsive beam under longtime illumination, and $\tau_{\mathrm{heat}} = \rho_c/k$ reflects the characteristic time for heat exchange between photothermal-responsive beam and environment. Note that the larger τ_{heat} indicates the longer time required for attaining the limited temperature difference T_{Limit} of the photothermally-responsive LCE cantilever.

Nondimensionalization. In this system, two time scales are present: inertial characteristic time $\tau_{\mathrm{inertial}} = \sqrt{ml^3/3\Pi}$ and heat time scale $\tau_{\mathrm{heat}} = \rho_c/k$. Actually, τ_{inertial} is the reciprocal of the

natural angular frequency ω_0. By introducing the dimensionless parameters $\bar{w} = w/l$, $\bar{w}_{\mathrm{L}} = w_{\mathrm{L}}/l$, $\bar{t} = t/\tau_{\mathrm{inertial}}$, $\bar{\beta} = \beta\tau_{\mathrm{inertial}}/m$, $\bar{w}_0 = w_0/l$, and $\bar{\tau}_{\mathrm{heat}} = \tau_{\mathrm{heat}}/\tau_{\mathrm{inertial}}$, Eq. (6) can be rewritten as

$$\frac{\mathrm{d}^2\bar{w}(t)}{\mathrm{d}\bar{t}^2} = -\bar{\beta}\frac{\mathrm{d}\bar{w}(t)}{\mathrm{d}\bar{t}} + \bar{w}_L(t) - \bar{w}(t). \tag{13}$$

Then, Eq. (11) and (12) can also be rewritten as,

in illuminated state, i.e.$\bar{w} \leq \bar{w}_0$:

$$\bar{w}_L(\bar{t}) = \bar{P}\left(1 - e^{-\bar{t}/\bar{\tau}_{\mathrm{heat}}}\right), \tag{14}$$

in non-illuminated state, i.e. $\bar{w} > \bar{w}_0$:

$$\bar{w}_L(\bar{t}) = \bar{P}e^{-\bar{t}/\bar{\tau}_{\mathrm{heat}}}, \tag{15}$$

where $\bar{P} = \lambda P$, with $\lambda = \frac{A\eta}{kl}$ denoting the deflection coefficient of light-driven bending. Notably, $\bar{P}$ denotes the dimensionless limit deflection of photothermally-responsive LCE cantilever under longtime illumination.

Eqs. (13-15) governing the vibration of the single oscillator under light beam. The typical values of materials properties and geometric parameters for the following calculations are listed in Supplementary Table 1.

Supplementary Table 1 Material properties and geometric parameters.

Parameter	Definition	Value	Unit
l	Length of the LCE cantilever	2.5	cm
w_0	Critical deflection (delay distance)	5	mm
$\bar{\beta}$	Dimensionless damping coefficient	0.8	/
τ_{inertial}	Inertial characteristic time	0.015	s
τ_{heat}	Heat time scale	0.3	s
λ	Deflection coefficient of light-driven bending	0.0026	/mW
P_0	Laser power	0~1000	mW

Amplitude and period of a single oscillator. By numerically solving the governing equations (13)-(15), Figure 34 plots the time history of the displacement of the baffle, for different laser powers. In the calculation, we set l =2.5 cm, w_0= 5 mm, $\bar{\beta}$ = 0.8, τ_{inertial} = 0.015 s, τ_{heat} = 0.3

s, and λ=0.0026 /mW. It is shown that the amplitude of self-oscillation increases monotonously with increasing laser power. Figure 35 plots the dependence of self-oscillation amplitude on the laser power. The result shows that there exists a Hopf bifurcation between the static state and the self-oscillation state. Above the Hopf bifurcation point P_{crit}=100 mW, the amplitude of self-oscillation increases with the laser power. In addition, the theoretical prediction aligns with the experimental results, as shown in Figure 35.

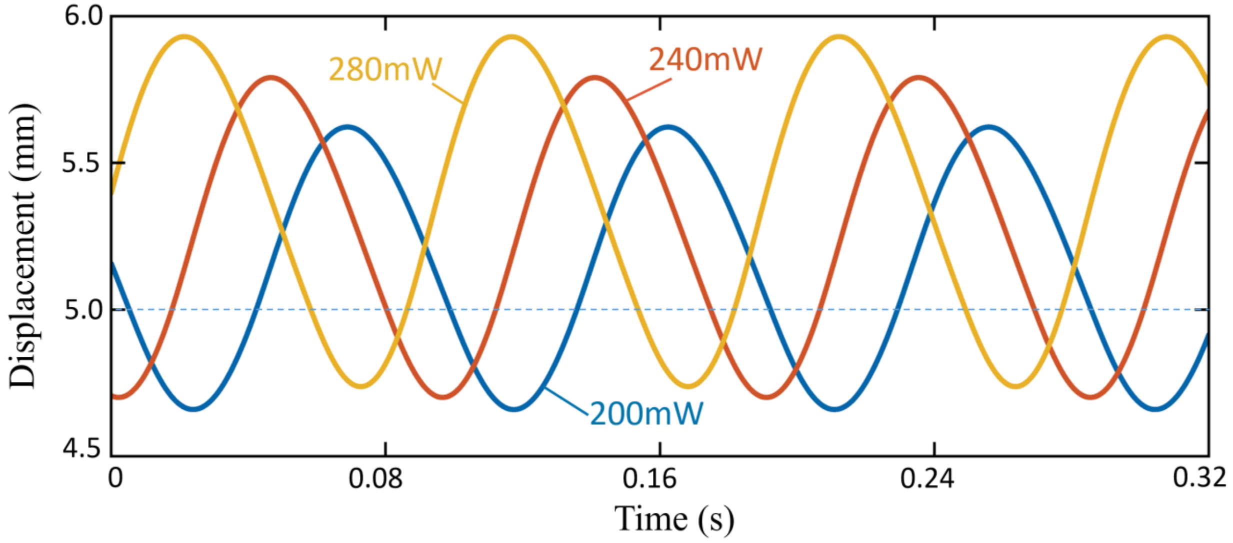

Figure 34. Numerical results. Time history of the displacement of the mass block, for three different laser powers P=200mW, 240mW, and 280mW. In the simulation, we set l =2.5 cm, w_0= 5mm, $\bar{\beta}$ = 0.8, τ_{inertial} = 0.015 s, τ_{heat} = 0.3 s, and λ=0.0026 /mW.

Meanwhile, the numerical results show that the period is mainly determined by the natural period T_0 = 0.1 s. This theoretical prediction is consistent with the experiment as shown in Figure 3. From Eq. (13), the period of the self-oscillation is derived as,

$$T = \frac{2\pi\tau_{\text{inertial}}}{\sqrt{1-\frac{\bar{\beta}^2}{4}}}. \tag{16}$$

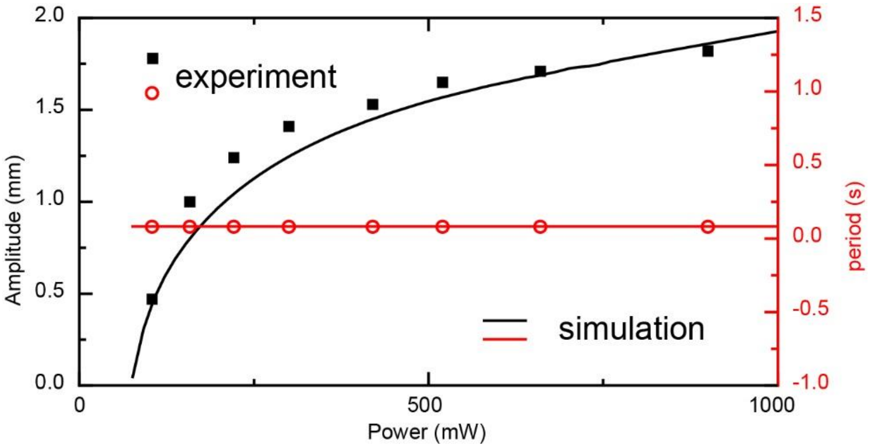

Figure 35. Dependences of amplitude and period of single oscillator on the laser power. In the simulation, we set l = 2.5 cm, w_0= 5 mm, $\bar{\beta}$ = 0.8, τ_{inertial} = 0.015 s, τ_{heat} = 0.3 s, and λ =0.0026/mW. Laser spot size: 2 mm.

2.2 Coupled oscillators.

Governing equations of coupled oscillators. Two light beams are used to connect the two LCE beams which are coupled, as shown in Figure 36. Light beam (+) goes near the edge of the baffle on the LCE cantilever (-), and light beam (-) is initially blocked by the baffle on the LCE cantilever (+) (Figure 36a). When light beam (+) hits the LCE cantilever (+), it unblocks light beam (-) (Figure 36b). Then, light beam (-) excites LCE cantilever (-) blocking light beam (+) (Figure 36c). As a result, LCE cantilever (+) relaxes toward its original position, blocking light beam (-) (Figure 36d), and LCE cantilever (-) relaxes toward the original position to unblock light beam (+) (Figure 36e). Then, light beam (+) hits the LCE cantilever (+), and unblocks light beam (-) (Figure 36b). The system returns to the previous state (Figure 36b), and a new cycle starts.

The end deflections of LCE cantilevers (-) and (+) are denoted by $w_-(t)$ and $w_+(t)$. The laser powers of light beams on LCE cantilevers (-) and (+) are denoted by P_- and P_+. For two coupled oscillators, the governing equations are written as

$$\frac{\mathrm{d}^2\bar{w}_-(t)}{\mathrm{d}\bar{t}^2} = -\bar{\beta}\frac{\mathrm{d}\bar{w}_-(t)}{\mathrm{d}\bar{t}} + \bar{w}_{\text{L-}}(t) - \bar{w}_-(t), \tag{17}$$

$$\frac{\mathrm{d}^2\bar{w}_+(t)}{\mathrm{d}\bar{t}^2} = -\bar{\beta}\frac{\mathrm{d}\bar{w}_+(t)}{\mathrm{d}\bar{t}} + \bar{w}_{\text{L+}}(t) - \bar{w}_+(t), , \tag{18}$$

where

$$\bar{w}_{\text{L-}}(\bar{t}) = \begin{cases} \bar{P}_{-}\left(1 - e^{-\frac{\bar{t}}{\tau_{\text{heat}}}}\right), \bar{w}_{+} > \bar{w}_{0+} \\ \bar{P}_{-} e^{-\frac{\bar{t}}{\tau_{\text{heat}}}}, \bar{w}_{+} < \bar{w}_{0+} \end{cases}, \tag{19}$$

$$\bar{w}_{\text{L+}}(\bar{t}) = \begin{cases} \bar{P}_{+}\left(1 - e^{-\frac{\bar{t}}{\tau_{\text{heat}}}}\right), \bar{w}_{-} < \bar{w}_{0-} \\ \bar{P}_{+} e^{-\frac{\bar{t}}{\tau_{\text{heat}}}}, \bar{w}_{-} > \bar{w}_{0-} \end{cases}, \tag{20}$$

in which, $\bar{w}_{0-}$ and $\bar{w}_{0+}$ denotes the on/off transition critical deflections of LCE cantilevers (-) and (+), respectively. The typical values of materials properties and geometric parameters for the following calculations are listed in Supplementary Table 2.

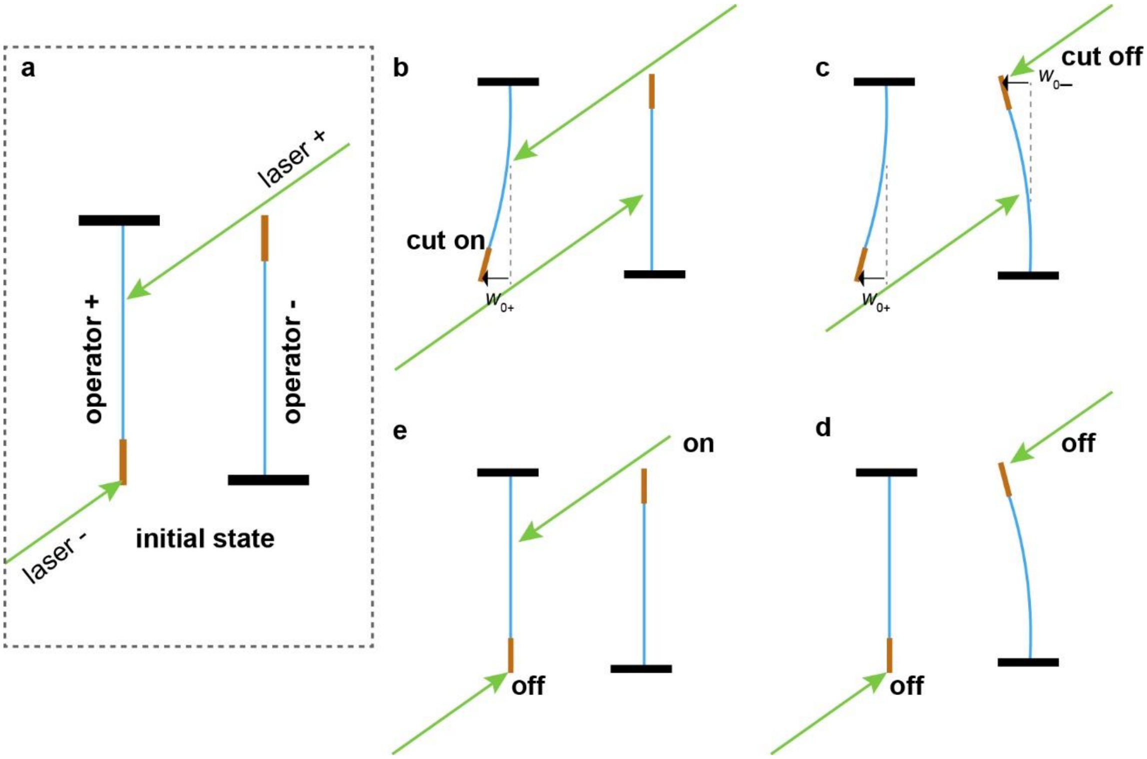

Figure 36. Schematics of two coupled oscillators. Two light beams are used to couple two LCE operators (a). Beam (+) goes near the edge of the baffle on the LCE (-), and beam (-) is initially blocked by the LCE (+) (a). When beam (+) hits the LCE (+), it unblocks beam (-) (b). Then, beam (-) excites LCE (-) blocking beam (+) (c). LCE (+) relaxes to its original position, blocking beam (-) (d), and LCE (-) relaxes to unblock light beam (+) (e). Then, light beam (+) hits the LCE (+), and unblocks light beam (-) – the system returns to the previous state (b). The mass of the LCE cantilever is ignored.

Supplementary Table 2 Material properties and geometric parameters for coupled oscillators.

Parameter	Definition	Value	Unit
l_-	Length of the LCE cantilever -	2.5	cm
l_+	Length of the LCE cantilever +	2.5	cm
w_{0-}	Critical deflection of the cantilever (delay distance) -	0~5	mm
w_{0+}	Critical deflection of the cantilever (delay distance) +	0~5	mm
$\bar{\beta}$	Dimensionless damping coefficient	0.8	/
τ_{inertial}	Inertial characteristic time	0.015	s
τ_{heat}	Heat time scale	0.3	s
λ_-	Deflection coefficient of the LCE cantilever -	0.0017	/mW
λ_+	Deflection coefficient of the LCE cantilever +	0.001	/mW
P_-	Laser power of laser beam (-)	0~1000	mW
P_+	Laser power of laser beam (+)	0~1000	mW

Delay mechanism of two coupled oscillators. By solving Eqs. (17)-(20), Figure 37 plots the time histories of the two coupled oscillators. It is shown that there exists a time delay t_d between cut-off position and the equilibrium position, which is much different from the single oscillator discussed above. In the single oscillator, there is no time delay between cut-off position and the equilibrium position.

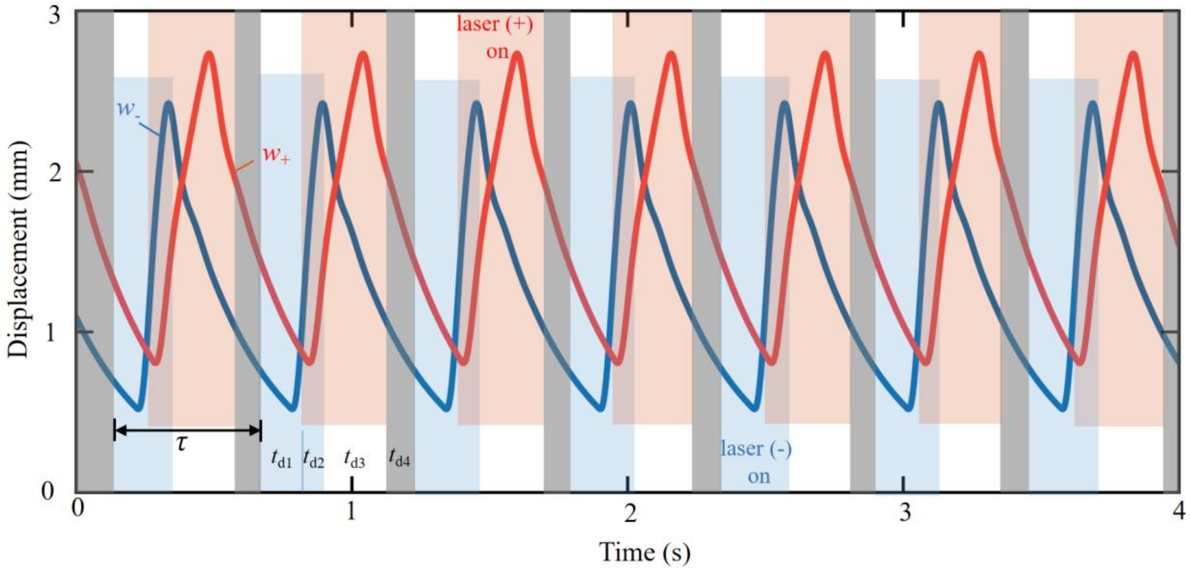

Figure 37. Time histories of two coupled oscillators. There exists time delay t_d in the oscillations of the two coupled oscillators. In the computation, we set l_- = 2.5 cm, l_+ = 2.5 cm, w_{0-} = 1.5 mm, w_{0+} = 1 mm, $\bar{\beta}$ = 0.8, τ_{inertial} = 0.015s, τ_{heat} = 0.3 s, P_- = 176 mW, P_+ = 200 mW, $\lambda_- = 0.0017$ /mW, $\lambda_+ = 0.001$ /mW.

Amplitude and period of two coupled oscillators. Figure 38 plots the dependence of the amplitude and period of the coupled oscillators on the laser power P_-. There exists a Hopf bifurcation between the static state and oscillation state. For laser power P_- smaller than the Hopf bifurcation point P_{crit}=100 mW, the LCE cantilever - bending is not enough to cut off the light beam +, and the circle cannot evolve. The prediction is qualitatively consistent with the experimental results.

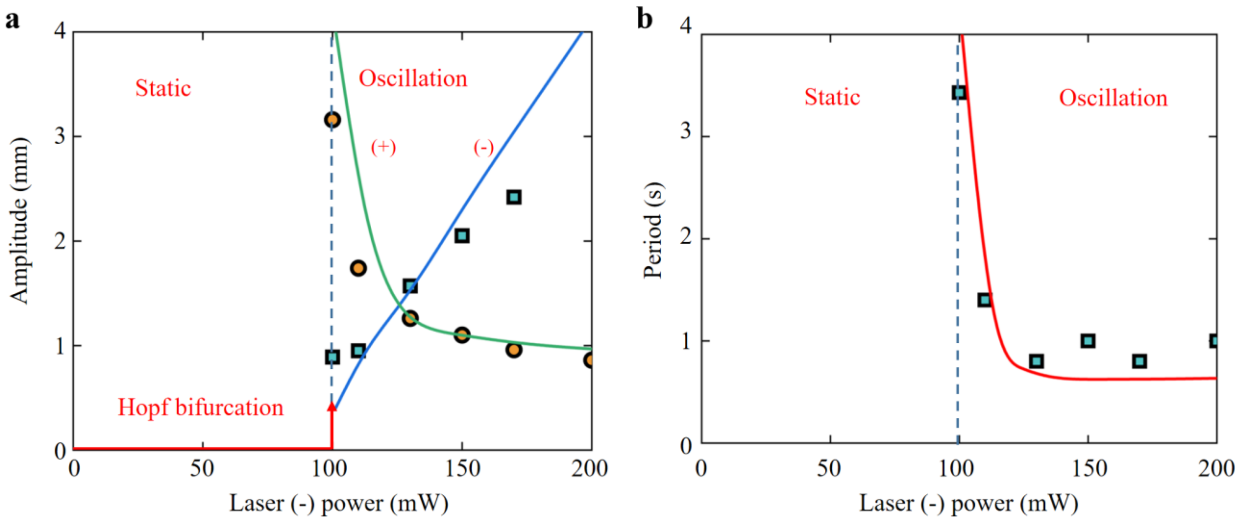

Figure 38. Simulated results for coupled oscillators. Amplitude (a) and period (b) of two coupled oscillators as a function of laser power (-). In the computation, we set $l_- = 2.5$ cm, $l_+ = 2.5$ cm, $w_{0-} = 4.25$ mm, $w_{0+} = 4.37$mm, $\bar{\beta} = 0.8$, $\tau_{\text{inertial}} = 0.015$s, $\tau_{\text{heat}} = 0.3$ s, $P_+ = 180$ mW, $\lambda_- = 0.0017$ /mW, $\lambda_+ = 0.001$ /mW. Spot size for laser (+), 2 mm, size for laser (-), 3 mm. Dots: experimental results. Solid lines: theoretical predictions.

Increase in one beam's power leads to the amplitude elevating of the operator such a beam directly excites on, and a decrease in the amplitude of the other operator that controls the beam, as shown in Fig. 38 and 39.

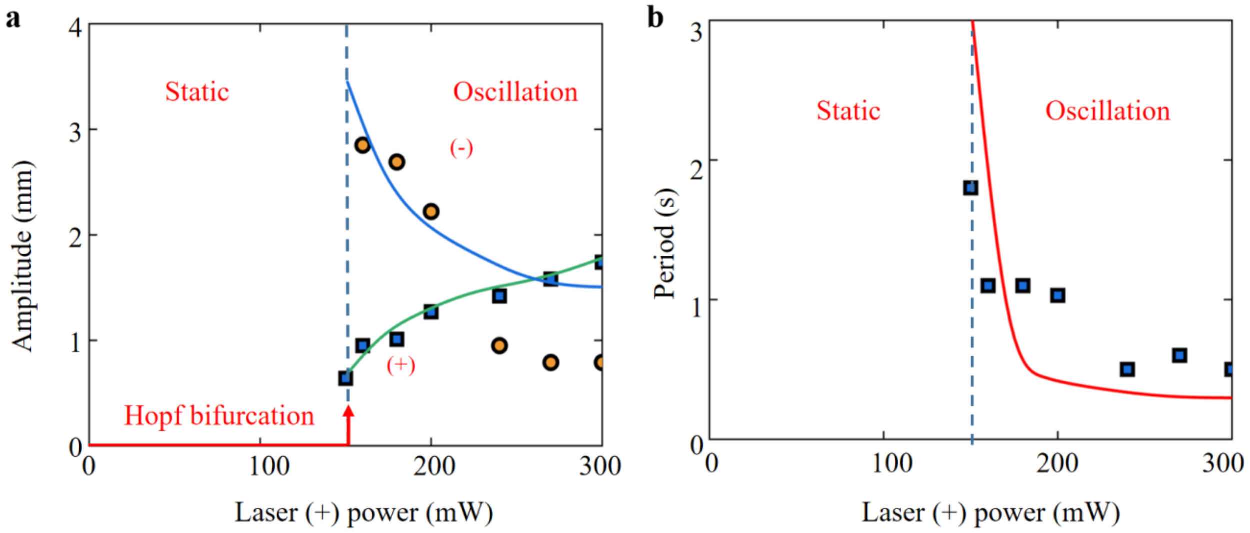

Figure 39. Simulated results for coupled oscillators. Amplitude (a) and period (b) of two coupled oscillators as a function of laser power (+). In the computation, we set $l_- = 2.5$ cm, $l_+ = 2.5$ cm, $w_{0-} = 3$ mm, $w_{0+} = 3.75$ mm, $\bar{\beta} = 0.8$, $\tau_{\text{inertial}} = 0.015$s, $\tau_{\text{heat}} = 0.3$ s, $\lambda_- = 0.0017$ /mW, $\lambda_+ = 0.001$ /mW. Spot size for laser (+), 2 mm, size for laser (-), 3 mm. Dots: experimental results. Solid lines: theoretical predictions.

Meanwhile, Figures 38 and 39 show that by increasing laser power P_{-}, the amplitude of LCE cantilever (-) increases while the amplitude of cantilever (+) decreases. In addition, the period decreases with the increase of the laser power P_{-}. These results can be understood from the effect of laser power on the time delay. The time delay decreases with increasing laser power, due to quick deflection and a faster cut-OFF action, thus shortening the oscillation cycle.

From Figure 37, it can be seen that the period is approximately four times the time delay. For given critical deflection $\bar{w}_{-0} \approx \bar{w}_{+0} \approx \bar{w}_0$ and $\bar{P}_{-} \approx \bar{P}_{+} \approx \bar{P}$, the time delay can be approximately estimated from Eqs. (19) and (20) as $\Delta t \approx -\tau_{\mathrm{heat}} \ln\left(1 - \frac{\bar{w}_0}{\bar{P}}\right)$, and the period can be estimated as

$$T \approx -4\tau_{\mathrm{heat}} \ln\left(1 - \frac{\bar{w}_0}{\bar{P}}\right). \tag{21}$$

From Eq. (21), it can also be understood that the period decreases with increasing laser power.

2.3 Comparison between single and coupled oscillators

The change of oscillation period. The oscillation behaviour of the single self-oscillator is dominated by the mechanical resonance, of which frequency is governed by Eq. (16). Taking $\omega_0 = 1/\tau_{\text{inertial}} = \sqrt{3\Pi/ml^3}$, Π=$E_Y I_i$, Π is bending stiffness, E_Y, Young's modulus, I_i, second moment of area, l, m are the length and mass of the operator, respectively. The oscillation frequency of single self-oscillator can be written as,

$$f_s = \frac{\sqrt{\frac{3E_Y I_i}{ml^3}\left(1 - \frac{\beta^2 l^3}{12 E_Y I_i m}\right)}}{2\pi} \qquad (22)$$

Variation of the material's mechanical properties such as change of Young's modulus (referred to tensile testing in Supplementary Fig. 3d), and geometric parameter, *i.e.* the second moment of area changes directly the self-oscillation frequency of the single oscillator.

After being coupled with two laser beams the coupled oscillator's frequency is no longer determined by the resonance frequency of single oscillator. The large difference in oscillation behaviour lies in a distance-dependent delay process in the feedback coupled oscillator. The oscillation behaviour is predicted by Eq. (21). Restoring from nondimensionalization treatment by taking $\bar{w}_0 = w_0/l$, $\bar{P} = \lambda P$, with $\lambda = \frac{A\eta}{kl}$, ($A$ is the light-driven bending coefficient, η is the energy absorption coefficient, k is the heat transfer coefficient), the oscillation period can be written as,

$$T \approx -4\tau_{\text{heat}} \ln\left(1 - \frac{k w_0}{A\eta P}\right) \qquad (23)$$

Change of oscillation behaviour. Changing the actuator material alters a complex combination of parameters, including τ_{heat}, k, A, η. For example, reducing the crosslinking density lowers the nematic-to-isotropic phase transition temperature (T_{ni}) from above 300 °C as observed in this study (Supplementary Fig. 3c), to around 100 °C (Ref. 2), which effectively increases A. Increasing the sample thickness, on the other hand, can reduce A while also changing the thermal capacity and increasing the value of τ_{heat}.

However, with the mechanical properties held constant, variations in delay distance w_0 – the minimum deflection distance of the baffle required to trigger the cut-on/-off action – can significantly influence the oscillation behavior. Figure 40a presents simulated results showing that increasing w_0 at one of the operators reduces the oscillation frequency and affects the waveform.

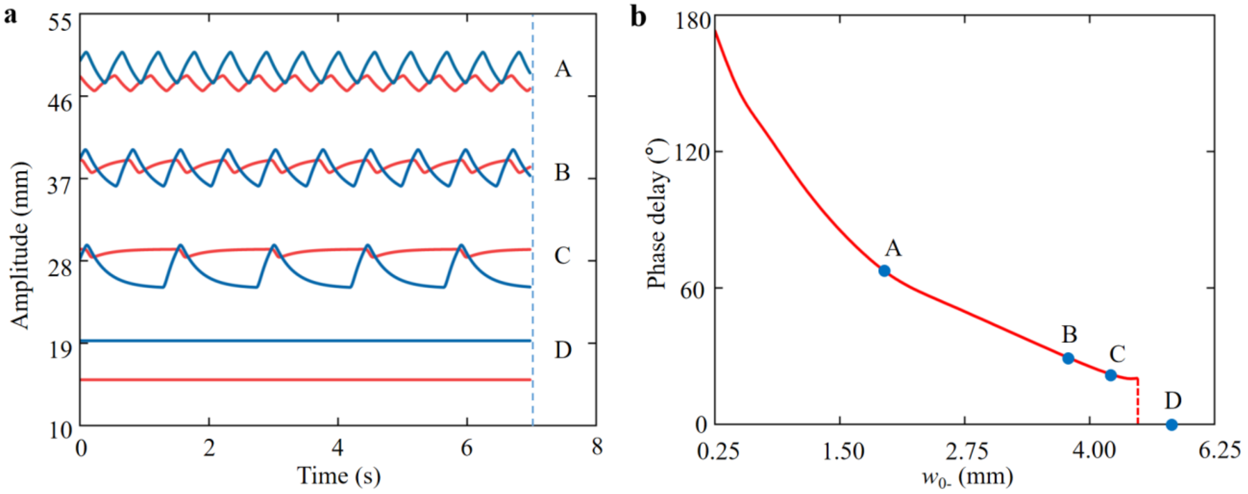

Figure 40 Influence of delay distance on the oscillation property. (a) Waveform and (b) phase delay of two coupled oscillators by change of the delay distance in the negative operator (w_{0-}). In the computation, we set l_- = 2.5 cm, l_+ = 2.5 cm, w_{0+} = 4.37 mm, $\bar{\beta}$ = 0.8, τ_{inertial} = 0.015s, τ_{heat} = 0.3 s, P_- = 106 mW, P_+ = 360 mW, λ_- = 0.0017 /mW, λ_+ = 0.001 /mW.

The theoretical analysis also indicates that the change of w_0 also influences the phase difference between two coupled oscillators. Figure 40b presents simulated results showing that an increase of w_0 reduces the phase delay from near 180 to 20° at high w_0. However, in the experiments, the phase delay highly depends on the irradiation power and sensitive to environmental fluctuations. Comparison between experimental and simulated results is challenging in this study.

Equivalence model. A light-fueled self-oscillator is a mechanical structure that oscillates under a constant light field. In both single-oscillator and coupled-oscillator systems, a negative feedback loop governs the self-oscillation process. Figure 41 illustrates this mechanism through an equivalent model, highlighting the kinetics of the negative operator.

In the single self-oscillator, the mechanism is relatively straightforward. The beam deflects from its equilibrium position to a critical distance w_0, at which point it blocks the incident light that excites itself. This interruption acts as a negative feedback signal, temporarily turning off the excitation and later on turning on it, sustaining continuous oscillation.

In the coupled oscillator system, the beam also deflects to a critical distance and blocks the incident

light at w_0. However, this blocking does not directly turn off the light exciting the same operator. Instead, it first extinguishes the light exciting the positive operator. Once the positive operator is turned off, it in turn blocks the other light beam, thereby switching off the light exciting the negative operator. This requires the baffles on both operators always travel for a longer distance than w_0, introducing an additional time delay dependent on the deflection distance and the photothermal response time of the material. A similar delayed mechanism occurs when the baffle returns to switch the light back on.

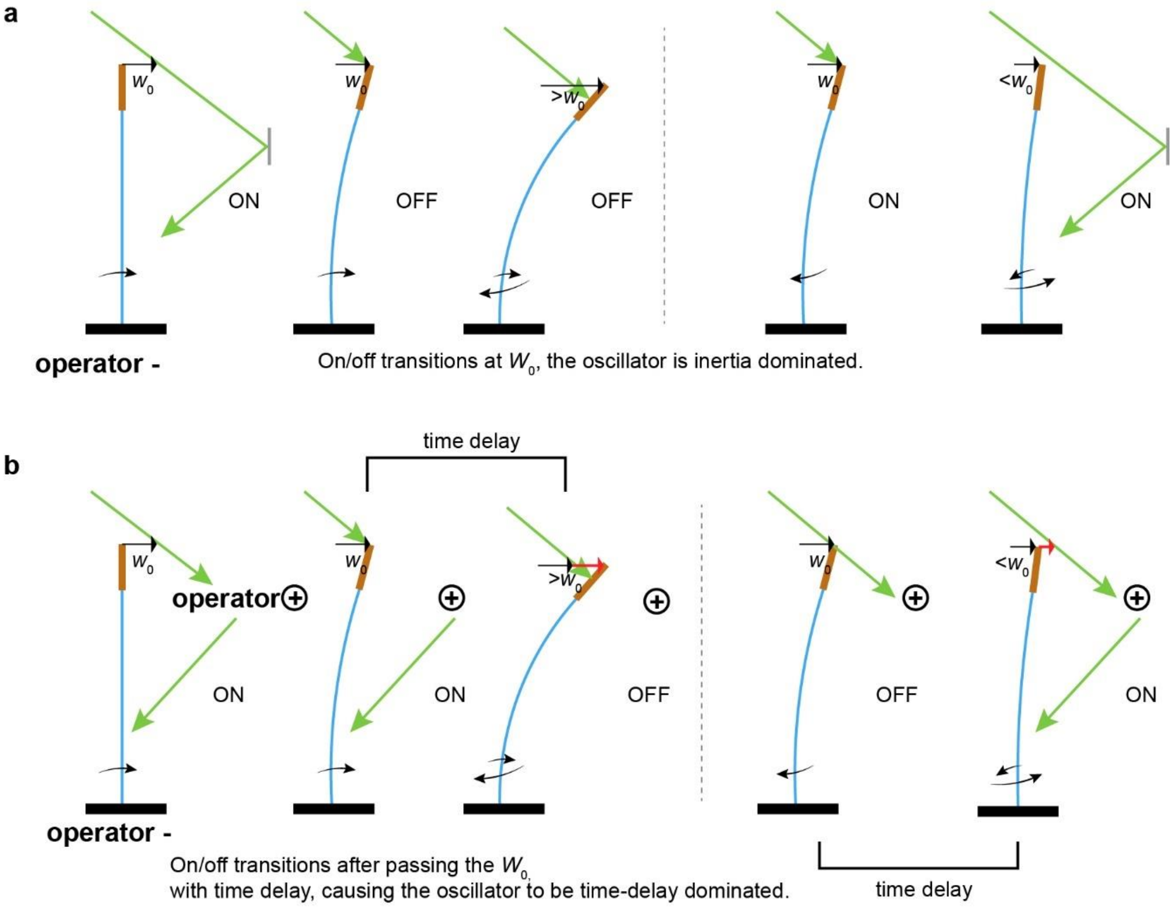

Figure 41 Equivalence model for coupled oscillators. Kinetics of (a) single self-oscillator and (b) coupled oscillators with time delay.

2.4 Sensing and control

For robotic applications, the light communication concept provides a method for remote sensing and electrical control of soft actuators over long distances. Figure 42a illustrates that the deformation of operator 1 affects the transmission intensity of the nearby light beam. The opening and closing actions of the baffle in operator 1 transmit an on-off signal of light intensity. By using a photodiode detector, the deformation signal from operator 1 is converted into an electrical signal, which is then received at the location of operator 2, as shown by the displacement and voltage data in Figure 42b. This enables remote sensing, such that, for example, a wind disturbance affecting operator 1 can be detected by the variation in the electrical signal at the position of operator 2 (Figure 42c). Note that both the nonlinear response of the photodiode and the scattered light from the nearby aluminium baffle affect the waveform, transforming it from an ideal rectangular shape to one with a domed top. More detailed remote sensing data, including long- and short-period perturbations from wind and mechanical triggers, can be found in Fig. 43.

Conversely, a tethered voltage signal can induce deformation of operator 1 via an electromagnetic coil, while a light beam can independently control operator 2 over a long distance. The working principle is illustrated in Figure 42d, with setup images provided in Fig. 44. When a voltage is applied to the electromagnetic coil, the magnetic field generates an attractive force that deforms operator 1, thereby activating the light beam, as shown by the displacement and light power data in Figure 42e. The transmitted beam then excites operator 2, causing deformation. Thus, the voltage signal near operator 1 can remotely control the deformation of operator 2 (Figure 42f). A negative voltage creates a propelling force that deforms operator 1 in the opposite direction, causing the light transmission to close. Control data based on the negative voltage signal is shown in Fig. 45. Robotic control can also be achieved by modulating the signal with different bandwidths, as demonstrated in Figure 42g and Fig. 46.

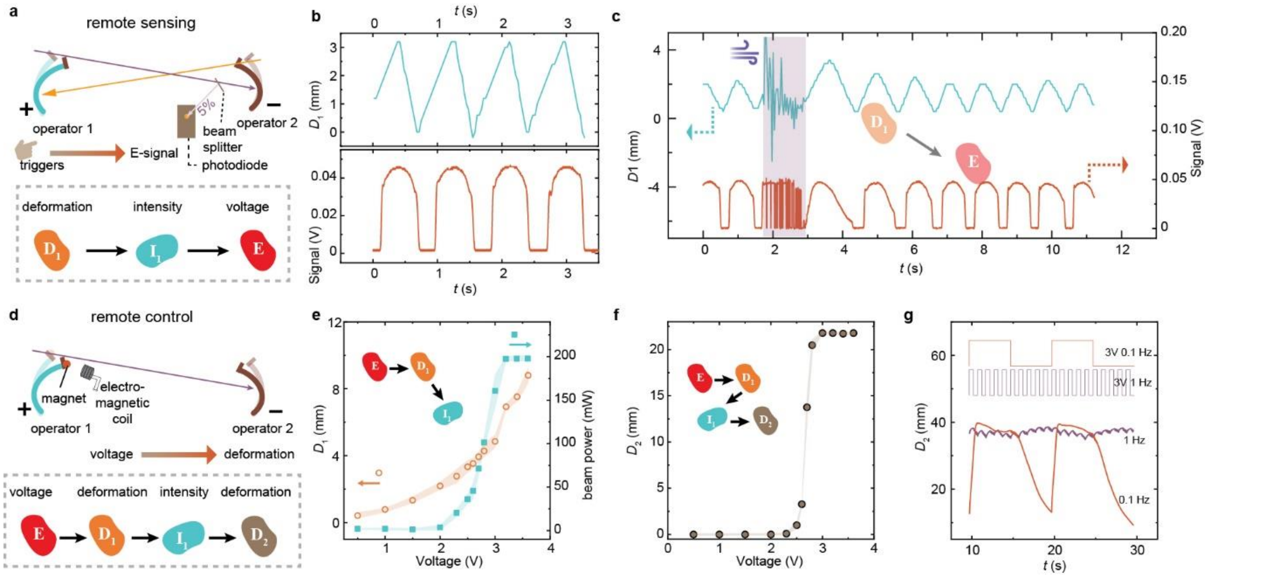

Figure 42. Remote sensing and robotic control. (a) Top: Schematic illustration of the two-unit network connected via two light beams. Bottom: Mechanism of signal transmission, showing how material deformation is converted into an electrical signal at a remote distance. (b) Displacement data from operator 1 and the corresponding electric signal received near operator 2. (c) Detection of wind disturbance at operator 1 and subsequent signal transition observed at the detector near operator 2. (d) Top: Schematic of the setup for remote deformation control. Bottom: Explanation of the remote-control mechanism. (e) Displacement of operator 1 and transmitted light power at various applied voltages. (f) Displacement of operator 2 in response to voltage changes applied to the electromagnetic coil near operator 1. (g) Modulation of operator 2 via the application of a square wave signal voltage to the electromagnetic coil. E, electric voltage generated by a photodiode detector or voltage applied to an electromagnetic coil. I_1, intensity of beam 1. D_1, displacement of operator 1. D_2, displacement of operator 2. (b, c) Laser 1: 532 nm, 256 mW, 3 mm. Laser 2: 532 nm, 200 mW, 2 mm. LCE sample dimensions: 24 × 2 × 0.1 mm^3, baffle size: 5 × 20 × 0.01 mm^3. (e-g) Laser: 532 nm, 200 mW, 2 mm.

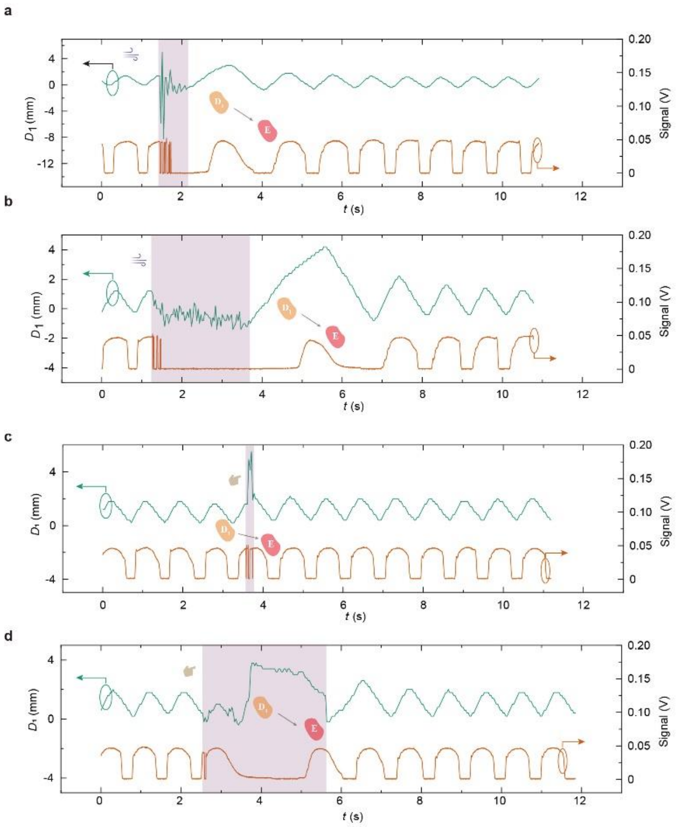

Figure 43. Remote sensing. Oscillation data of the coupled oscillators in response to external wind disturbance applied to operator 1 for (a) short and (b) long durations, and the received electrical signal at a position near operator 2. Oscillation data of the coupled oscillators in response to external mechanical disturbance applied to operator 1 for (c) short and (d) long durations, and the received electrical signal at a position near operator 2. Laser 1: 532 nm, 256 mW, 3 mm. Laser 2: 532 nm, 200 mW, 2 mm. LCE sample dimensions: $24 \times 2 \times 0.1$ mm^3, baffle size: $5 \times 20 \times 0.01$ mm^3. Scale bar: 2 cm.

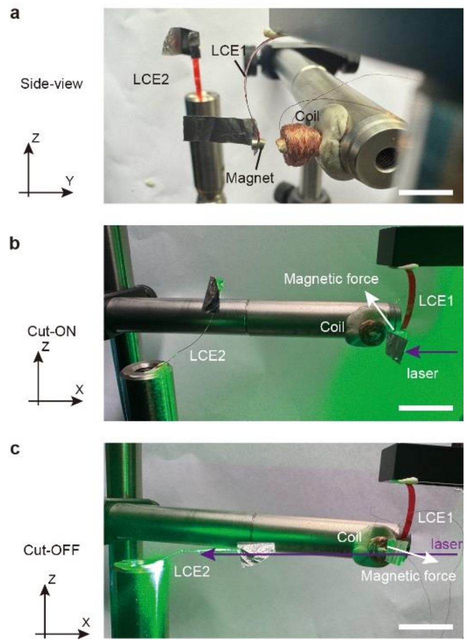

Figure 44. Photograph of the electric control system. (a) Side view showing the light-coupled components and electromagnetic coil. Front view images of the system with (b) the 'cut-ON' state activated by a positive voltage and (c) the 'cut-OFF' state activated by a negative voltage signal. Two magnets are mounted on the baffle of one component near the coil. Scale bar is 1 cm. Magnet: Neodymium 50 magnet, 2mm diameter × 1mm thick, 23mg weight. The coil is made of copper wire with 1,000 turns.

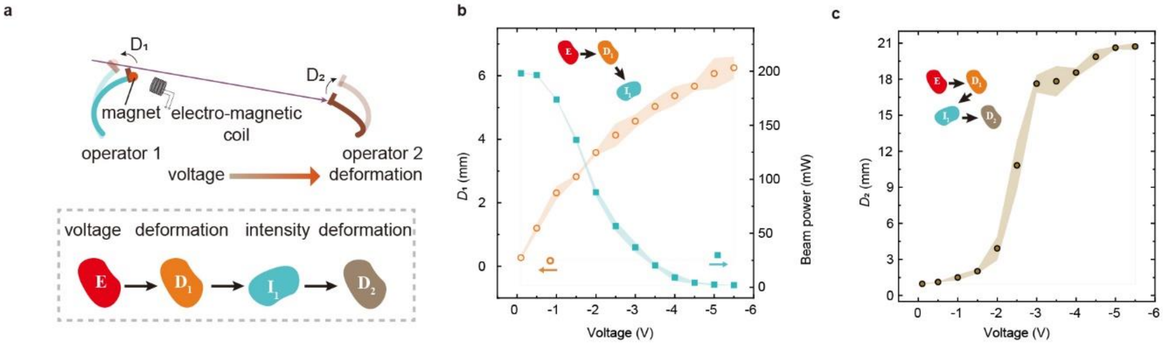

Figure 45. Remote control system. (a) Top: Schematic of the setup for remote deformation control. Bottom: Explanation of the remote-control mechanism. (b) Displacement of operator 1 and transmitted light power at various applied voltages. (c) Displacement of operator 2 in response to voltage changes applied to the electromagnetic coil near operator 1. *E*, electric voltage applied to the electromagnetic coil. I_1, intensity of beam 1. D_1, displacement of operator 1. D_2, displacement of operator 2. Laser 1: 532 nm, 200 mW, 2 mm. LCE sample dimensions: $24 \times 2 \times 0.1$ mm^3, baffle size: $5 \times 20 \times 0.01$ mm^3.

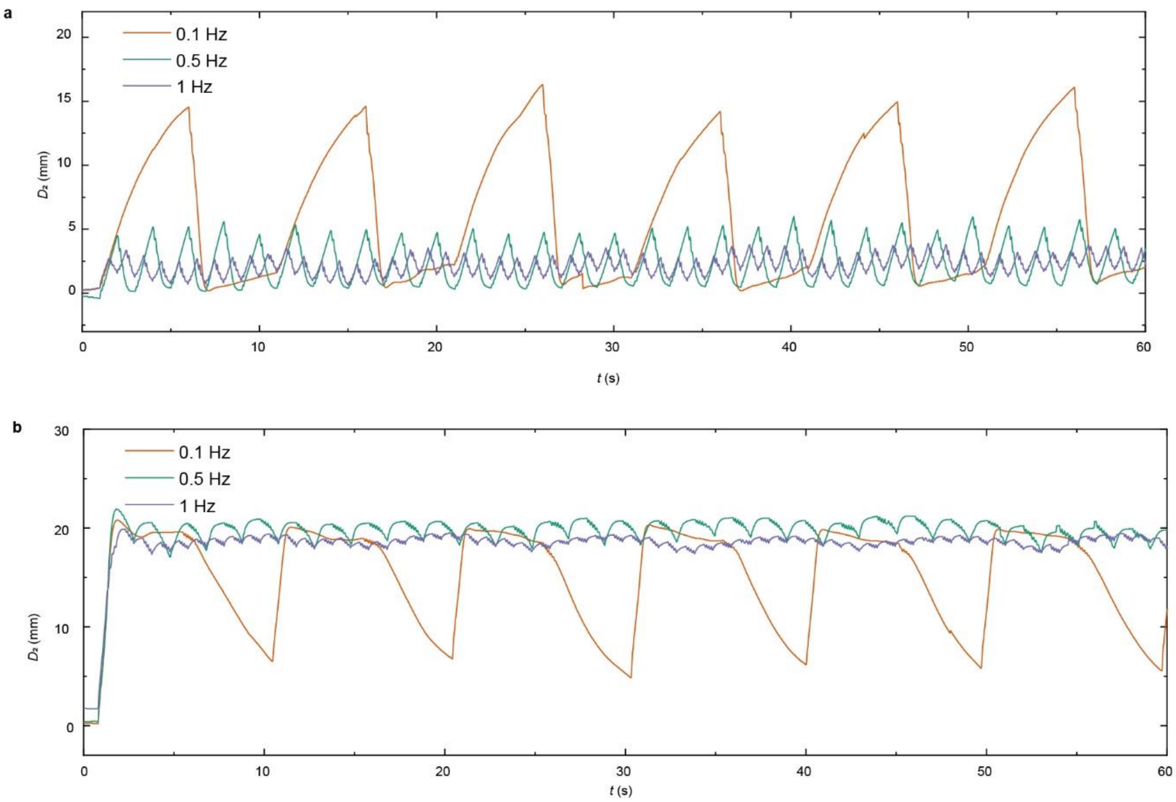

Figure 46. Remote control data. Modulation of operator 2 via the application of a square wave voltage signal. Voltage: (a) 0 to -5 V, (b) 0 to 3 V. 50% duty cycle. D_2, displacement of operator 2.

2.5 Scalability – a future plan.

In mechanics[3] the resonance frequency of a vibrating cantilever scales with L^{-1} (L is the characteristic length of the object), thermal gradient scales with L^{-1}, mass and heat capacity with L^3 – all indicating the fact that as the size is reduced, the natural frequency, photothermal heating and cooling (when ceasing the light) speeds increase. Along with the increased in-plane unit density (scales with L^{-2}), thus we believe that micro-structured LCEs can provide a systematic approach to obtain fast response, high sampling rate and integration for micro-chip devices.

Two-photon (2-p) laser fabrication can produce light-controlled LCE and 3D photo-resin elements[4,5] in microscopic scale. To program the deformation modes in soft LCE, a magnetic field (> 0.5 T)[6] created by placing two magnets side-by-side on a rotatable aluminum frame, can be used to control the LC director field, endowing 4D laser fabrication. Conventional methods such as rubbed surface coating and homeotropic alignment layer can also be adopted to align the LCE structures in cases where the deformation behavior does not require pixel-by-pixel control. Laser fabrication will also generate resin-based 3D rigid elements for baffles, LCE supports and waveguides[7] to build the network.

Below, we introduce the systematization approach for enhancing network complexity. The material network is composed of multiple units. Each unit is based on a light-deformable LCE finger and an optical baffle, as shown in Fig. 47.

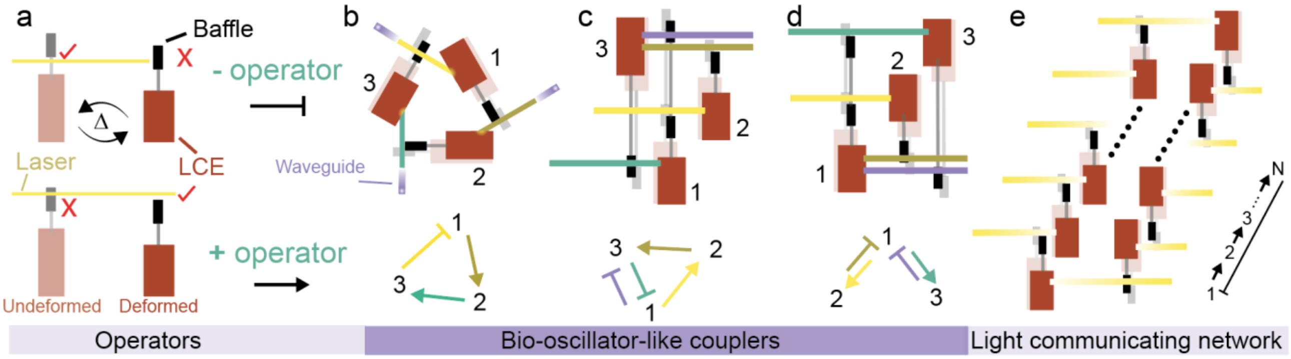

Figure 47. Bio-inspired communicating network. (a) Baffle-LCE structure functioned as "-" and "+" operators. Structural design (top) and self-regulating motif (bottom) for bio-mimic oscillators built on (b) basic negative feedback, (c) incoherent feedforward and (d) chaos behavioral loops. (e) Design of a light communicating network composed of *N* operator units.

Depending on the excitation beam position (from the waveguide) with respect to the baffle, the LCE fingers can either deform to block the light (Fig. 47a, top) or switch on the light stimuli (Fig.

47a, bottom), thus functioning as negative (–) and positive (+) operators, respectively. Sequential operators will be assembled at micro-scale and equipped with excitation waveguides. The network will be looped up to form the self-oscillating network. Note that the unit number can be larger than 3, and at least one of the operators should be "–" sign to close the feedback loop that dictates the oscillation.

The interaction between these operator units can be systematized to form a communicating network. This is based on the facts that (i) the 3D-fabricated waveguides can arbitrarily establish the linkages between any operators (either "–" or "+"), and (ii) each LCE finger can be equipped with multiple baffles and act as multi-functioned operator (i.e. "–" & "+"). Three examples are given to present, (1) three-component negative feedback-loop (basic form of bio-oscillator, Fig. 47b), (2) incoherent feedforward loop (additional negative feedback for added robustness, Fig. 47c), and (3) two negative feedback to the same activator (chaos, Fig. 47d), all taken the inspiration from biochemical oscillators[1].

3. References

1. Novák, B. & Tyson, J. J. Design principles of biochemical oscillators. *Nat. Rev. Mol. Cell Bio.* **9**, 981-991 (2008).
2. Yoon, H-H, Kim, D-Y, Jeong, K-U, Ahn, S-K. Surface aligned main-chain liquid crystalline elastomers: tailored properties by the choice of amine chain extenders, Macromolecules 2018, 51, 1141−1149.
3. M. Wautelet, *Scali*ng laws in the macro-, micro- and nanoworlds. Eur. J. Phys. 2001, 22, 601.
4. H. Zeng, D. Martella, P. Wasylczyk, G. Cerretti, J.-C. G. Lavocat, C.-H. Ho, C. Parmeggiani, D. S. Wiersma, High-Resolution 3D Direct Laser Writing for Liquid-Crystalline Elastomer Microstructures. Adv. Mater. 2014, 26, 2319.
5. M. Malinauskas, M. Farsari, A. Piskarskas, S. Juodkazis, Ultrafast laser nanostructuring of photopolymers: A decade of advances. Phys. Rep. 2013, 533, 1.

6. B. Ni, G. Liu, M. Zhang, M. Tatoulian, P. Keller, M.-H. Li, Customizable Sophisticated Three-Dimensional Shape Changes of Large-Size Liquid Crystal Elastomer Actuators. ACS Appl. Mater. Interfaces 2021, 13, 54439.
7. S. Nocentini, F. Riboli, M. Burresi, D. Martella, C. Parmeggiani, D. S. Wiersma, Three-Dimensional Photonic Circuits in Rigid and Soft Polymers Tunable by Light. ACS Photonics 2018, 5, 3222.
