# Supplementary material for: Light-mediated communication in responsive materials ranging from individual self-oscillators to feedback-driven network
Source: Nat Commun. 2025 Nov 20;16:11315. doi: 10.1038/s41467-025-66395-3 (PMC12722257; doi:10.1038/s41467-025-66395-3)
Supplement: Supplementary file 11 — Description of Additional Supplementary Files [file 41467_2025_66395_MOESM11_ESM.pdf]

# Description of Additional Supplementary Files

**Supplementary Movie 1. Coupled self-oscillators.**
This real-time video shows continuous oscillations of two coupled operators. The black markings facilitate position tracking during the oscillation. Laser 1 (right to left): 532 nm, 150 mW, 2 mm spot size. Laser 2 (left to right): 635 nm, 200 mW, 3 mm spot size. LCE actuator dimensions: 24 × 2 × 0.1 $mm^3$. Baffle dimension: 5 × 20 × 0.01 $mm^2$. The optical pathway follows the same design in Fig. 2c.

**Supplementary Movie 2. Coupling between two isolated self-oscillators.**
This real-time video shows continuous oscillations of two coupled operators with a screen in the middle. The laser beams are guided around the screen and reflected onto the sample through mirrors. Laser 1 (exciting on left sample): 532 nm, 150 mW, 2 mm spot size. Laser 2 (exciting on right sample): 635 nm, 300 mW, 3 mm spot size. LCE actuator dimensions: 24 × 2 × 0.1 $mm^3$. Baffle dimension: 5 × 20 × 0.01 $mm^2$. The optical pathway follows the same design in Fig. 3a.

**Supplementary Movie 3. Coupling between two self-oscillators through optical fibers.**
This real-time video shows continuous oscillations of two coupled operators through optical fibers. Laser 1 (exciting on upper sample): 532 nm, 256 mW, 3 mm spot size. Laser 2 (exciting on bottom sample): 532 nm, 200 mW, 2 mm spot size. LCE actuator dimensions: 24 × 2 × 0.1 $mm^3$. Baffle dimension: 5 × 20 × 0.01 $mm^2$. The samples and connections to fibers are shown in Fig. 3h.

**Supplementary Movie 4. Cascading transition.**
This real-time video shows the cascading transition from ON to OFF state and then from OFF to ON state. All Laser: 532 nm, 100 mW, 2 mm spot size. LCE actuator dimensions: 16 × 2 × 0.1 $mm^3$. Baffle dimension: 4 × 12 × 0.01 $mm^2$. The optical pathway follows the same design in Fig. 4e.

**Supplementary Movie 5. Dual rhythm in self-oscillation.**
This real-time video shows the mechanical trigger induced transition between two oscillation frequencies. Laser 1 excited on operator 1: 532 nm, 180 mW, 2 mm; Laser 2 spot on operator 2: 532 nm, 180 mW, 2 mm; Laser 3 spot on operator 1: 532 nm, 160 mW, 2 mm. LCE fiber dimensions: 1.6 cm in length and 1 mm in diameter. Baffle dimension: 4 × 12 × 0.01 $mm^2$. The optical pathway follows the same design in Fig. 4h.

**Supplementary Movie 6. Coupled network composed of three units.**
This video shows continuous oscillations of three coupled operators. The laser beams are guided through optical fibers. All laser beams: 532 nm, 320 mW, 2 mm spot size at the sample positions. LCE actuator dimensions: 24 × 2 × 0.1 $mm^3$. Baffle dimension: 5 × 20 × 0.01 $mm^2$. The movie is played with 4× accelerated speed. The optical pathway is schematically shown in Supplementary Figure 22c.

**Supplementary Movie 7. Coupled network composed of four units.**
This video shows continuous oscillations of four coupled operators. The laser beams are guided through optical fibers. All laser beams: 532 nm, 320 mW, 2 mm spot size at the sample positions. LCE actuator dimensions: 24 × 2 × 0.1 $mm^3$. Baffle dimension: 5 × 20 × 0.01 $mm^2$. A red coloured filter is used to block laser wavelengths for video recording. The movie is played with 4× accelerated speed. The optical

pathway is schematically shown in Supplementary Fig. 22b.

**Supplementary Movie 8. The coupling between a thermometer and LCE actuator.**
This video shows continuous oscillations of the coupled system. Laser 1 (exciting on LCE): 532 nm, 44 mW, 2 mm spot size. Laser 2 (exciting on thermometer): 532 nm, 930 mW, 3 mm spot size. LCE actuator dimensions: $24 \times 2 \times 0.1$ mm$^3$. Baffle dimension: $5 \times 20 \times 0.01$ mm$^2$. The movie is played with 2× accelerated speed. The optical pathway is schematically shown in Supplementary Fig. 28.

**Supplementary Movie 9. The coupling between the paraffin and LCE actuator.**
This video shows continuous oscillations of the coupled system. Laser 1 (exciting on LCE): 532 nm, 80 mW, 1.2 mm spot size. Laser 2 (exciting on paraffin): 532 nm, 1440 mW, 2 mm spot size. LCE actuator dimensions: $24 \times 2 \times 0.1$ mm$^3$. Baffle dimension: $5 \times 20 \times 0.01$ mm$^2$. The movie is played with 16× accelerated speed. The optical pathway follows the same design in Supplementary Fig. 29.
